# Effect of hydroxychloroquine on pregnancy outcome in patients with SLE: a systematic review and meta-analysis

**DOI:** 10.1136/lupus-2024-001239

**Published:** 2024-10-30

**Authors:** Qingmiao Zhu, Jiayu Wang, Qice Sun, Zhijun Xie, Rongqun Li, Zi Yang, Ziyu Song, Kepeng Yang, Ting Zhao

**Affiliations:** 1 School of Basic Medical Sciences, Zhejiang Chinese Medical University, Hangzhou, Zhejiang, China; 2 The First School of Clinical Medicine, Zhejiang Chinese Medical University, Hangzhou, Zhejiang, China; 3 The Second Affiliated Hospital of Zhejiang Chinese Medical University, Hangzhou, Zhejiang, China; 4 Key Laboratory of Chinese medicine rheumatology of Zhejiang Province, Research Institute of Chinese Medical Clinical Foundation and Immunology, College of Basic Medical Science, Zhejiang Chinese Medical University, Hangzhou, Zhejiang, China; 5 The Third School of Clinical Medicine, Zhejiang Chinese Medical University, Hangzhou, Zhejiang, China

**Keywords:** Lupus Erythematosus, Systemic, Autoimmune Diseases, Antirheumatic Agents

## Abstract

**Objective:**

Hydroxychloroquine (HCQ) is an antimalarial drug employed in the treatment of systemic lupus erythematosus (SLE). Prior studies reported inconsistent results regarding the association between HCQ use during pregnancy and adverse pregnancy outcomes. This study aimed to evaluate the impact of HCQ on pregnancy-related outcomes in women with SLE.

**Methods:**

We conducted a systematic search for studies associating pregnancy outcomes with HCQ use in PubMed, EMBASE, Cochrane Library, Web of Science, CNKI, Wanfang database and VIP from inception to 22 September 2022. Random or fixed effect models were used to estimate the pooled effect based on I^2^ measurement of heterogeneity.

**Results:**

Twenty-one studies were included, encompassing 929 and 1031 patients in HCQ and non-HCQ groups, respectively. We found that HCQ use was significantly associated with reduced risks of Systemic Lupus Erythematosus Disease Activity Index (SLEDAI) scores (second trimester: mean difference (MD) −1.80, 95% CI −2.46 to –1.13; third trimester: MD −2.30, 95% CI −3.31 to –1.29), flare (OR 0.57, 95% CI 0.33 to 0.97), preterm birth (OR 0.57, 95% CI 0.46 to 0.72), intrauterine growth retardation (IUGR) (OR 0.48, 95% CI 0.31 to 0.72), gestational hypertension (OR 0.19, 95% CI 0.08 to 0.42), pre-eclampsia (OR 0.46, 95% CI 0.29 to 0.72). In contrast, a positive correlation was observed between full-term birth and HCQ use (OR 2.01, 95% CI 1.52 to 2.65). However, the result for disease flare exhibited high heterogeneity (p=0.01, I^2^=59%). In addition, publication bias was detected in the meta-analysis of full-term birth using the Egger’s test.

**Conclusions:**

This meta-analysis offers a comprehensive assessment of the relationship between disease activity, pregnancy-related outcomes and HCQ use, providing supportive evidence for the therapeutic effectiveness of HCQ in pregnant women with SLE.

**PROSPERO registration number:**

CRD42022374468.

**WHAT IS ALREADY KNOWN ON THIS TOPIC**

- The utilisation of hydroxychloroquine (HCQ) during pregnancy remains controversial.

**WHAT THIS STUDY ADDS**

- HCQ appears to positively impact the reduction of disease activity in pregnant women suffering from SLE.
- HCQ contributes to a decline in the incidence of preterm birth and intrauterine growth retardation (IUGR) in women with SLE.
- The risk of gestational hypertension and pre-eclampsia decreases with the use of HCQ in women with SLE.

**HOW THIS STUDY MIGHT AFFECT RESEARCH, PRACTICE OR POLICY**

- This study offers evidence that supports the effectiveness of HCQ during pregnancy, thereby assisting in clinical decision-making.

## Introduction

 SLE is a multisystem autoimmune disease with diverse clinical manifestations. The pathogenesis of SLE is still not clear and may be associated with genetic, environmental, immune abnormalities and other factors. In recent years, the global incidence and prevalence of SLE have reached 5.14 (1.4–15.13) per 100 000 and 43.7 (15.87–108.92) per 100 000, among which there are certain differences in gender and age. According to statistics, adult females are more susceptible to SLE.^1^ Women with SLE face many challenges during pregnancy. SLE may lead to adverse pregnancy outcomes and complications, such as preterm birth, miscarriage, stillbirth, intrauterine growth retardation (IUGR), small for gestational age (SGA) and pre-eclampsia. Also, pregnant women with SLE have a higher risk of disease flares. With the improvement of medical care and the management of lupus pregnancy, although pregnancy remains a high-risk endeavour for female patients with SLE, patients can have successful pregnancies and deliveries under strict monitoring and treatment.^2 3^ According to the European Alliance of Associations for Rheumatology (EULAR) guidelines and related literature, it is stated that some medications to alleviate lupus such as mycophenolate mofetil, cyclophosphamide, methotrexate and leflunomide should be discontinued before conception due to their potential teratogenicity and the increased risk of miscarriage.^4^ However, some medications such as hydroxychloroquine (HCQ), azathioprine (AZA), sulfasalazine and ciclosporin A (CsA) are considered safe, especially HCQ, which is recommended for continued use throughout the pregnancy.^5^

HCQ is a 4-aminoquinoline derivative antimalarial drug with anti-inflammatory, immunomodulatory and anti-infective effects.^6^ It has been observed that HCQ administration during pregnancy offers beneficial effects for both the mother and the fetus. From the maternal perspective, the use of HCQ prevents disease flares and achieves remission or lower disease activity,^7^,^10^ while reducing the risk of various pregnancy complications such as pre-eclampsia.^11 12^ From the perspective of pregnancy outcomes, HCQ demonstrates an obvious advantage in improving adverse pregnancy outcomes including preterm birth, miscarriage and IUGR.^813^,^16^

Currently, HCQ serves as a fundamental therapy for SLE and is employed during pregnancy. Nonetheless, the effectiveness and safety of this treatment modality require thorough evaluation. Existing guidelines and literature^47^,^18^ suggest that HCQ usage is advantageous in preventing recurrence and reducing the occurrence of adverse pregnancy outcomes. However, it has also been reported that HCQ might increase the risk of certain adverse pregnancy outcomes, such as miscarriage and congenital malformations.^19^,^21^ Consequently, we performed an updated comprehensive meta-analysis to assess the safety and effectiveness of HCQ in pregnancy among women with SLE concerning disease activity, pregnancy outcomes, fetal outcomes and pregnancy complications.

## Materials and methods

The systematic review was conducted following the Preferred Reporting Items for Systematic Reviews and Meta-Analysis (PRISMA), with the protocol registered in the International Prospective Register of Systematic Reviews (PROSPERO). The registration number is CRD42022374468. For the meta-analysis of published observational studies and randomised controlled trials (RCTs), a formal ethics approval was not required.

### Date sources and search strategy

PubMed, EMBASE, Cochrane Library, Web of Science, Chinese National Knowledge Infrastructure database (CNKI), Wanfang database, Chinese Scientific Journal database (VIP) were systematically searched from inception to 22 September 2022. Our search strategy combined four separate search strings: hydroxychloroquine, pregnancy, lupus erythematosus, systemic and pregnancy outcome. We used both Medical Subject Headings (MeSH) terms and free-text words to evaluate the effect of HCQ on adverse pregnancy outcomes in patients with lupus. The complete search strategies are detailed in online supplemental material A. No restrictions were placed on language.

### Eligibility criteria

Two investigators (QZ, JW) independently screened titles and abstracts. Disagreements that remained unresolved were referred to a third reviewer (QS).

Studies were included in the meta-analysis if they met the following criteria: (1) case-control studies, cohort studies or RCTs; (2) comparison of two groups of patients: those receiving HCQ during pregnancy (HCQ group) and those not receiving it during pregnancy (non-HCQ group); (3) documentation of pregnancy and fetal outcomes.

The following studies were excluded from the meta-analysis: (1) studies without a group of participants not using HCQ; (2) duplicate studies; (3) reviews, comments, animal experiments, case reports, literature with only abstracts, meta-analyses and unrelated studies; (4) studies where all relevant outcome data for the meta-analysis were missing and could not be obtained or inferred.

### Data extraction and quality assessment

Data were independently extracted from eligible studies by two reviewers (QZ, JW) using a predesigned extraction form, and disagreements were resolved by consensus or by a third reviewer (QS). The extracted data were categorised into three main parts: (1) study inclusion details, including the first author and publication year; (2) characteristics of eligible studies, including study type, number of participants using and not using HCQ, disease duration, average age, intervention/exposure factors, HCQ dose and medication use; (3) related outcome indicators, including SLE activity (flare, Systemic Lupus Erythematosus Disease Activity Index (SLEDAI)), pregnancy outcomes (full-term birth, preterm birth, miscarriage, stillbirth), fetal outcomes (fetal distress, IUGR, low birth weight, SGA) and pregnancy complications (gestational hypertension, pre-eclampsia, gestational diabetes mellitus).

Considering that the included articles were all cohort studies or case-control studies, two investigators (QZ, JW) independently assessed the quality of the included articles using the Newcastle-Ottawa Scale (NOS).

### Statistical analysis

For dichotomous outcomes, we calculated the pooled risk ratio (OR) and 95% CI, and for continuous variables, we choose the mean difference (MD) as effect size and 95% CI were calculated. The Cochrane Q test and I^2^ tests were used to determine between-study heterogeneity. Low heterogeneity was indicated by an I²≤50% and high heterogeneity by an I²>50%. A fixed effect model was applied to studies with I²≤50% and a random effect model for I²>50%. When heterogeneity was high, subgroup analysis or sensitivity analysis was performed to identify the sources of heterogeneity. Sensitivity analysis was also used to assess the robustness of the results by excluding each study individually. Additionally, Egger’s tests and funnel plots were employed to detect publication bias. Statistical significance was defined as p value <0.05, and the data were analysed using Review Manager V.5.3 and Stata V.17.

## Results

### Study selection and study characteristics

**Figure 1:**
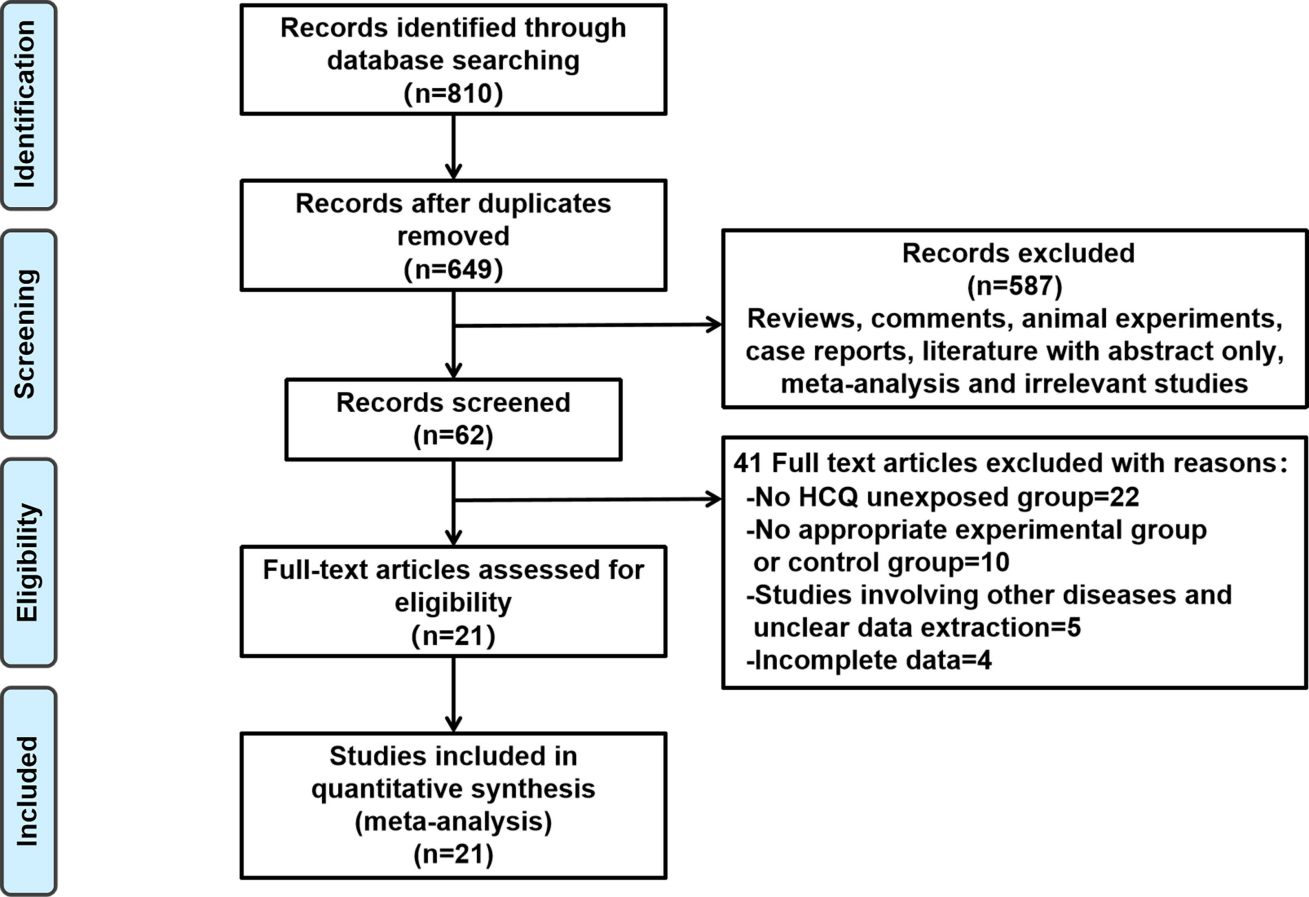
Flow diagram. HCQ, hydroxychloroquine.

We implemented a comprehensive search strategy to retrieve 810 relevant documents. After eliminating duplicates, we screened the remaining literature based on their titles and abstracts, ultimately selecting 62 for further assessment. On reviewing the full texts, 41 articles were excluded, resulting in 21 studies^1416 22^,^40^ that met the inclusion criteria (see figure 1). These studies included 4 case-control studies and 17 cohort studies, encompassing a total of 929 pregnancies exposed to HCQ and 1031 pregnancies not exposed to HCQ. Fourteen of these studies reported HCQ dosages ranging from 200 to 400 mg/day. Additional medications involved in the studies were corticosteroids, immunosuppressants such as CsA and AZA, low molecular weight heparin, aspirin, anticoagulants and intravenous immunoglobulin. The included studies reported on 16 SLE activity, 21 pregnancy outcomes, 15 fetal outcomes and 8 pregnancy complications. The basic characteristics of the studies were detailed in online supplemental material E.

### Assessment of the methodological quality of the literature

According to the NOSNOS, the scores for 21 studies varied between 5 and 8, indicating a fair quality assessment. The methodological quality assessment results are presented in online supplemental material B.

### Synthesis of results (forest plots of each meta-analysis with study-specific effect size and weight)

#### SLE activity

Nine studies examined SLE flares during pregnancy, involving 437 pregnancies in the HCQ group and 551 pregnancies in the non-HCQ group (table 1). High between-study heterogeneity was observed (p=0.01, I^2^=59%), thus a random effect model was used for meta-analysis. The results showed a lower rate of SLE flares in the HCQ group compared with the non-HCQ group (OR 0.57, 95% CI 0.33 to 0.97) (online supplemental material F)

**Table 1:** Summary of meta-analysis of SLE activity level

SLE activity	Studies	Pooled OR/MD (95% CI)	P value	I^2^ (%)	P value for heterogeneity
Flare	9	0.57 (0.33 to 0.97)*	0.04	59	0.01
SLEDAI: First trimester	7	−0.05 (−0.44 to 0.33)†	0.78	0	0.98
SLEDAI: Second trimester	7	−1.80 (−2.46 to −1.13)†	<0.01	64	0.01
SLEDAI: Third trimester	7	−2.30 (−3.31 to −1.29)†	<0.01	89	<0.01

Pooled OR/MD: Pooled ORs/mean difference for associations between HCQ use and SLE activity levels.

* OR.

† MD.

MDmean differenceORodds ratioSLEDAISystemic Lupus Erythematosus Disease Activity Index

Seven studies reported on SLEDAI in pregnant women with SLE across all three trimesters (table 1). (1) First trimester: No heterogeneity (p=0.98, I^2^=0%). Fixed effect model. No statistical significance in SLEDAI score difference between the HCQ and non-HCQ groups (MD −0.05, 95% CI −0.44 to 0.33). (2) Second trimester: High heterogeneity (p=0.01, I^2^=64%). Random effect model. SLEDAI score significantly lower in the HCQ group compared with the non-HCQ group (MD −1.80, 95% CI −2.46 to –1.13). (3) Third trimester: High heterogeneity (p<0.01, *I*^*2*^=89%). Random effect model. SLEDAI score significantly lower in the HCQ group than the non-HCQ group (MD −2.30, 95% CI −3.31 to –1.29).

#### Pregnancy outcome

Thirteen studies involving 427 pregnancies in the HCQ group and 623 pregnancies in the non-HCQ group reported full-term birth (table 2). The study exhibited low heterogeneity (p=0.04, I^2^=46%), allowing for a meta-analysis using a fixed effect model. The analysis revealed a significantly higher rate of full-term birth in the HCQ group compared with the non-HCQ group (OR 2.01, 95% CI 1.52 to 2.65).

**Table 2:** Summary of meta-analysis of pregnancy outcome

Pregnancy outcome	Studies	Pooled OR (95%CI)	P value	I^2^ (%)	P value for heterogeneity
Full-term birth	13	2.01 (1.52 to 2.65)	<0.01	46	0.04
Preterm birth	20	0.57 (0.46 to 0.72)	<0.01	36	0.06
Miscarriage	8	1.26 (0.79 to 2.01)	0.33	43	0.09
Stillbirth	9	0.82 (0.46 to 1.46)	0.49	0	0.65

Pooled OR: Pooled ORs for associations between HCQ use and pregnancy outcomes

HCQhydroxychloroquineORodds ratio

Twenty studies involving 903 pregnancies in the HCQ group and 1005 pregnancies in the non-HCQ group reported outcomes related to preterm birth (table 2). The studies exhibited low heterogeneity (p=0.06, I^2^=36%), allowing for the conduct of a meta-analysis using a fixed effect model. The rate of preterm birth was significantly reduced in the HCQ group compared with the non-HCQ group (OR 0.57, 95%CI 0.46 to 0.72).

Eight studies containing 509 pregnancies in the HCQ group and 501 pregnancies in the non-HCQ group provided data concerning spontaneous miscarriage (table 2). The studies exhibited low between-study heterogeneity (p=0.09, I^2^=43%), thus a fixed effect model was used for the meta-analysis. The analysis revealed no significant difference in spontaneous miscarriage rates between the two groups (OR 1.26, 95% CI 0.79 to 2.01).

Nine studies involving a total of 535 pregnancies in the HCQ group and 575 pregnancies in the non-HCQ group reported incidents of stillbirths (table 2). There was no heterogeneity among the studies (p=0.65, *I*^*2*^=0%), thus a fixed effect model was used for the meta-analysis. The result showed no significant difference in the incidence of stillbirth between the HCQ group and the non-HCQ group (OR 0.82, 95% CI 0.46 to 1.46).

#### Fetal outcome

Five studies involving a total of 304 pregnancies in the HCQ group and 176 pregnancies in the non-HCQ group reported incidents of fetal distress (table 3). The study showed low heterogeneity between them (p=0.17, I^2^=37%), thus a fixed effect model was used for the meta-analysis. The analysis revealed no significant difference in the incidence of fetal distress between the two groups (OR 0.82, 95% CI 0.42 to 1.61).

**Table 3:** Summary of meta-analysis of fetal outcome

Fetal outcome	Studies	Pooled OR (95% CI)	P value	I^2^ (%)	P value for heterogeneity
Fetal distress	5	0.82 (0.42 to 1.61)	0.57	37	0.17
IUGR	11	0.48 (0.31 to 0.72)	<0.01	47	0.04
Low birth weight	4	0.43 (0.13 to 1.50)	0.19	74	<0.01
SGA	3	0.95 (0.60 to 1.53)	0.85	0	0.57

Pooled OR: Pooled ORs for associations between HCQ use and fetal outcomes. IUGR: A condition where fetal growth is restricted, often resulting in a birth weight below the 10th percentile for gestational age. Low birth weight: Birth weight less than 2500 g. SGA: Fetal weight <10th percentile for gestational age.

HCQhydroxychloroquineIUGRintrauterine growth retardationORodds ratioSGAsmall for gestational age

Eleven studies involving a total of 490 pregnancies in the HCQ group and 422 pregnancies in the non-HCQ group reported the incidence of IUGR (table 3). The study showed low heterogeneity between them (p=0.04, I^2^=47%), and a meta-analysis was conducted using a fixed effect model. The analysis revealed that the incidence of IUGR was significantly lower in the HCQ group compared with the non-HCQ group (OR 0.48, 95% CI 0.31 to 0.72).

Four studies involving 258 pregnancies in the HCQ group and 158 pregnancies in the non-HCQ group reported the number of infants born with low birth weight (table 3). High between-study heterogeneity was observed (p=0.009, I^2^=74%), prompting the use of a random effect model for meta-analysis. The result showed no significant difference in the incidence of low birthweight infants between the HCQ and non-HCQ group (OR 0.43, 95% CI 0.13 to 1.50).

Three studies involving 166 pregnancies in the HCQ group and 314 pregnancies in the non-HCQ group reported the occurrence of SGA (table 3). There was no heterogeneity among the studies (p=0.57, I^2^=0%), so a meta-analysis was conducted using a fixed effect model. The result showed no significant difference in the incidence of SGA between the HCQ and non-HCQ groups (OR 0.95, 95% CI 0.60 to 1.53).

#### Pregnancy complication

Four studies involving 176 pregnancies in the HCQ group and 181 pregnancies in the non-HCQ group reported maternal complications of gestational hypertension (table 4). The studies showed low heterogeneity between them (p=0.23, *I*^2^=30%), thus a meta-analysis was conducted using a fixed effect model. The result revealed a significantly lower incidence of gestational hypertension in the HCQ group compared with the non-HCQ group (OR 0.19, 95% CI 0.08 to 0.42).

**Table 4:** Summary of meta-analysis of pregnancy complication

Pregnancy complication	Studies	Pooled OR (95%CI)	P value	I^2^ (%)	P value for heterogeneity
Gestational hypertension	4	0.19 (0.08 to 0.42)	<0.01	30	0.23
Pre-eclampsia	8	0.46 (0.29 to 0.72)	<0.01	0	0.78
Gestational diabetes mellitus	3	0.75 (0.10 to 5.75)	0.78	61	0.08

Pooled OR: Pooled ORs for associations between HCQ use and pregnancy complications. Pre-eclampsia: Hypertension combined with proteinuria.

HCQhydroxychloroquineORodds ratio

Eight studies involving 476 pregnancies in the HCQ group and 448 pregnancies in the non-HCQ group reported the occurrence of pre-eclampsia (table 4). Since there was no heterogeneity between the studies (p=0.78, I^2^=0%), a meta-analysis was conducted using a fixed effect model. The result showed a significantly lower incidence of pre-eclampsia in the HCQ group compared with the non-HCQ group (OR 0.46, 95% CI 0.29 to 0.72).

Three studies encompassing 264 pregnancies in the HCQ group and 146 pregnancies in the non-HCQ group documented the incidence of gestational diabetes mellitus (table 4). High heterogeneity was observed between the studies (p=0.08, I^2^=61%), necessitating a random effect model for the meta-analysis. The analysis revealed no significant disparity in the incidence of gestational diabetes between the HCQ group and the non-HCQ group (OR 0.75, 95% CI 0.10 to 5.75).

### Sensitivity analysis and publication bias

Sensitivity analysis, involving the removal of individual studies one at a time, demonstrated that when each study was sequentially excluded and the meta-analysis was redone, the pooled effect sizes remained stable and reliable, not noticeable differing from the total effect sizes (see online supplemental material C). To assess potential publication bias in the included literature, funnel plots and Egger’s tests were employed. The funnel plot exhibited essential symmetry, and the p value for the Egger’s test exceeded 0.05, indicating the absence of publication bias. In contrast, asymmetry would suggest the presence of publication bias. Our findings revealed that the funnel plots of the relevant literature for outcome indicators were predominantly symmetrical, with p values greater than 0.05, indicating a lack of publication bias, except for full-term birth (p=0.003) (see online supplemental material D).

## Discussion

SLE is an autoimmune disease that affects multiple organs and is more common in women of childbearing age. For patients with lupus during pregnancy, medication use is subject to certain restrictions. HCQ, serving as an antimalarial and anti-rheumatic drug, possesses a complex mechanism of action that may induce pharmacological effects by intervening in lysosomal acidification, suppressing antigen presentation, inhibiting proinflammatory cytokine secretion, dampening autophagy and regulating multiple signalling pathways.^6 20^ In clinical practice, HCQ is extensively used for managing rheumatic immune diseases like SLE. Furthermore, established guidelines and literature stress the importance of administering HCQ to all patients with SLE, including pregnant women, unless there are specific contraindications or adverse effects.^4 5 21^

Our findings demonstrated that HCQ decreased the risk of relapse and mitigates disease symptoms during pregnancy and the postpartum period. In this study, we observed that HCQ therapy correlated with diminished disease activity, as assessed by SLEDAI scores. SLEDAI is the most commonly used scale for assessing SLE activity. Disease activity was comparable between the two groups in the first trimester. However, the SLEDAI score significantly decreased in the second trimester and the third trimester. This result aligns with the outcomes of several clinical studies.^7 8^ Despite this, a high degree of heterogeneity was detected in the meta-analysis of flare. Therefore, we performed a sensitivity analysis, which indicated that the study by Seo *et al* might contribute to the heterogeneity. Seo *et al* reported a significantly higher proportion of SLE flares in the HCQ group compared with the non-HCQ group (25% vs 11.3%, p=0.036). On further investigation, although the general characteristics of the two populations were generally consistent, the HCQ group had a significantly higher number of patients with antiphospholipid syndrome (APS) than the non-HCQ group (28.8% vs 8.5%, p=0.002). Li *et al* confirmed that APS can lead to maternal complications such as SLE flares.^41^ Nevertheless, the meta-analysis result remained beneficial even when excluding this study (OR 0.51, 95% CI 0.36 to 0.72). However, it remains important to consider the specific characteristics and conditions of individuals with SLE, including the presence of antiphospholipid antibodies, when evaluating the potential benefits of HCQ therapy.

In terms of pregnancy and fetal outcomes, our meta-analysis revealed that HCQ usage was associated with a decreased incidence of preterm birth and IUGR and contributes to full-term delivery of the fetus. Additionally, no significant associations were detected between the HCQ group and the non-HCQ group concerning miscarriage, stillbirth, fetal distress, low birthweight infants or SGA. These findings differed somewhat from those of Guillotin *et al*, who did not establish that HCQ prevents preterm birth or IUGR.^42^ It is also worth noting that the p value of Egger’s test for the full-term birth was 0.003, indicating the presence of publication bias. This suggests that the result of the meta-analysis on full-term birth should be interpreted with caution.

In addition, other important discoveries from this meta-analysis include the effectiveness of HCQ in preventing gestational hypertension and pre-eclampsia. The primary impact of SLE on women of childbearing age is an increased risk of pregnancy complications, including gestational hypertension and pre-eclampsia. Concurring with our findings, Duan *et al* analysed nine studies assessing HCQ’s role in preventing pregnancy complications, concluding that HCQ decreased the risk of gestational hypertension and pre-eclampsia in patients with SLE.^11^ It is noteworthy that some of the included studies^14 24 38^ had ambiguous definitions of pre-eclampsia, which may lead to confusion between the terms pre-eclampsia and eclampsia, potentially affecting the interpretation of the results. However, the sensitivity analysis, which excluded studies with unclear definitions, showed that the overall trend of the conclusions remained unchanged. Research indicated that HCQ might prevent pre-eclampsia by enhancing vascular endothelial function and immune response; however, the precise mechanism remains obscure and calls for further comprehensive investigation.^43^ Some studies have demonstrated that aspirin can effectively lower the incidence of pre-eclampsia.^44 45^ For the meta-analysis of pre-eclampsia, eight studies^1416 22 24^,^26 28 38^ were incorporated. Four of these studies^14 16 24 26^ reported that patients might have concurrently taken aspirin while on HCQ, while one study^25^ did not mention medication usage. After excluding these studies, no statistical difference was observed between the two groups (OR 0.52, 95 CI% 0.23, 1.15). Additionally, there is a certain relationship between parity and the occurrence of pre-eclampsia. Compared with multiparous women, primiparous women have a higher risk of pre-eclampsia. In the relevant studies we included, Seo *et al* and Do *et al* conducted stratified analyses of the study outcomes based on parity, while the other studies did not address this aspect. We cannot overlook the potential impact this may have on our study results.

Our study presents an updated and refined analysis in comparison to prior meta-analyses. Our research offers a more thorough evaluation, particularly in evaluating disease activity, pregnancy outcomes, fetal outcomes and pregnancy complications. Additionally, in contrast to the relevant meta-analysis by Clowse *et al*,^9^ which included relevant studies from 1995 to 2015, our study extends the search up to 22 September 2022, incorporating 14 eligible articles published between 2016 and 2022. Thus, our study is more timely and comprehensive.

However, we must acknowledge several limitations of our study. First, most of our studies were cohort studies, with the remainder being case-control studies, rather than RCTs. Cohort and case-control studies are subject to various biases and confounding factors that RCTs are specifically designed to minimise. A significant limitation of our study is the lack of RCTs. Future research should incorporate more RCTs to provide stronger evidence and clearer conclusions. Fortunately, the quality of the selected literature was satisfactory. Second, due to the lack of standardised dosing regimens, we were unable to assess the potential confounding effects of other medications such as steroids and aspirin. It has been reported that glucocorticoid may lead to adverse pregnancy outcomes.^46 47^ In addition, aspirin is often used to prevent pregnancy complications. The effects of other drugs on pregnancy cannot be ignored. Third, patients who did not comply with medical advice to take a regular dose of HCQ on time faced a higher risk of adverse pregnancy outcomes.^48^ However, we were unable to assess patient adherence using scientific methods. Fourth, current evidence suggests an association between the dose of HCQ and pregnancy-related outcomes.^49^ However, HCQ doses were not well-described or inconsistent in some of the included studies. Additionally, although HCQ was used during pregnancy in all studies included, the frequency and timing of HCQ use were not uniformly defined. Consequently, the influence of dose errors and variations in usage on outcomes could not be accurately assessed. Lastly, due to the unclear description of patients’ racial or ethnic background in the included study, subgroup analyses were difficult to perform. What we learnt from the literature is that region is a contributing factor to SLE recurrence in pregnant women, with Asian women tending to experience more relapses during pregnancy and the postpartum period.^50^ Thus, it is recommended that higher quality clinical trials be conducted in the future to validate our findings, with standardised medication dosages and regimens to eliminate potential confounding factors such as dosage and adherence.

In conclusion, HCQ demonstrates benefits in reducing the risk of recurrence, mitigating disease activity, promoting full-term birth and preventing preterm birth and IUGR. Furthermore, it lowers the risk of gestational hypertension and pre-eclampsia. Although there are limitations to consider, this study provides evidence supporting the safety and effectiveness of HCQ during pregnancy, thus aiding in clinical decision-making.

## supplementary material

10.1136/lupus-2024-001239 online supplemental file 1

10.1136/lupus-2024-001239 online supplemental file 2

10.1136/lupus-2024-001239 online supplemental file 3

10.1136/lupus-2024-001239 online supplemental file 4

10.1136/lupus-2024-001239 online supplemental file 5

10.1136/lupus-2024-001239 online supplemental file 6

## Data Availability

All data relevant to the study are included in the article or uploaded as supplementary information.
