# Supplementary material for: Effect of hydroxychloroquine on pregnancy outcome in patients with SLE: a systematic review and meta-analysis
Source: Lupus Sci Med. 2024 Oct 30;11(2):e001239. doi: 10.1136/lupus-2024-001239 (PMC11529578; doi:10.1136/lupus-2024-001239)
Supplement: online supplemental file 1 [file lupus-11-2-s001.pdf]

Supplementary material A: Search Terms

Exposures: Hydroxychloroquine	"Hydroxychloroquine"[Mesh] OR Oxychlorochin[Title/Abstract] OR Oxychloroquine[Title/Abstract] OR Hydroxychlorochin[Title/Abstract] OR Plaquenil[Title/Abstract] OR "Hydroxychloroquine Sulfate"[Title/Abstract] OR "Hydroxychloroquine Sulfate (1:1) Salt"[Title/Abstract]
Participants: Pregnancy	"Pregnancy"[Mesh] OR Pregnancies[Title/Abstract] OR Gestation[Title/Abstract] OR Gravidity[Title/Abstract] OR "Labor, Obstetric"[Title/Abstract] OR "Maternal-Fetal Exchange"[Title/Abstract] OR Parity[Title/Abstract] OR Parturition[Title/Abstract] OR Placentation[Title/Abstract] OR "Prenatal Nutritional Physiological Phenomena"[Title/Abstract] OR Pseudopregnancy[Title/Abstract]
Participants: SLE	"Lupus Erythematosus, Systemic"[Mesh] OR "Systemic Lupus Erythematosus"[Title/Abstract] OR "Lupus Erythematosus Disseminatus"[Title/Abstract] OR "Libman-Sacks Disease"[Title/Abstract] OR "Disease, Libman-Sacks"[Title/Abstract] OR "Libman Sacks Disease"[Title/Abstract] OR "Lupus Nephritis"[Title/Abstract] OR "Lupus Vasculitis, Central Nervous System"[Title/Abstract]
Outcome: APOs	"Pregnancy Outcome"[Mesh] OR "Pregnancy Outcomes"[Title/Abstract] OR "Outcome, Pregnancy"[Title/Abstract] OR "Outcomes, Pregnancy"[Title/Abstract] OR "Live Birth"[Title/Abstract] OR "Abortion, Spontaneous"[Title/Abstract] OR "Abortions, Spontaneous"[Title/Abstract] OR "Spontaneous Abortions"[Title/Abstract] OR "Spontaneous Abortion"[Title/Abstract] OR "Early Pregnancy Loss"[Title/Abstract] OR "Early Pregnancy Losses"[Title/Abstract] OR "Loss, Early Pregnancy"[Title/Abstract] OR "Losses, Early Pregnancy"[Title/Abstract] OR "Pregnancy Loss, Early"[Title/Abstract] OR "Pregnancy Losses, Early"[Title/Abstract] OR Miscarriage[Title/Abstract] OR Miscarriages[Title/Abstract] OR "Abortion, Tubal"[Title/Abstract] OR "Abortions, Tubal"[Title/Abstract] OR "Tubal Abortion"[Title/Abstract] OR "Tubal Abortions"[Title/Abstract] OR "Abortion, Habitual"[Title/Abstract] OR "Abortion, Incomplete"[Title/Abstract] OR "Abortion, Missed"[Title/Abstract] OR "Abortion, Septic"[Title/Abstract] OR "Abortion, Veterinary"[Title/Abstract] OR "Embryo Loss"[Title/Abstract] OR Stillbirth[Title/Abstract] OR "Premature birth"[Title/Abstract] OR "Birth, Premature"[Title/Abstract] OR "Births, Premature"[Title/Abstract] OR "Premature Births"[Title/Abstract] OR "Preterm Birth"[Title/Abstract] OR "Birth, Preterm"[Title/Abstract] OR "Births, Preterm"[Title/Abstract] OR "Preterm Births"[Title/Abstract] OR "Infant, Premature"[Title/Abstract]

	OR "Labor, Premature"[Title/Abstract] OR "Infant, Low Birth Weight"[Title/Abstract] OR "Low-Birth-Weight Infant"[Title/Abstract] OR "Infant, Low-Birth-Weight"[Title/Abstract] OR "Infants, Low-Birth-Weight"[Title/Abstract] OR "Low Birth Weight Infant"[Title/Abstract] OR "Low-Birth-Weight Infants"[Title/Abstract] OR "Low Birth Weight"[Title/Abstract] OR "Birth Weight, Low"[Title/Abstract] OR "Birth Weights, Low"[Title/Abstract] OR "Low Birth Weights"[Title/Abstract] OR "Infant, Small for Gestational Age"[Title/Abstract] OR "Infant, Very Low Birth Weight"[Title/Abstract] OR "Infant, Extremely Low Birth Weight"[Title/Abstract] OR "Fetal Macrosomia"[Title/Abstract] OR "Fetal Macrosomias"[Title/Abstract] OR "Macrosomias, Fetal"[Title/Abstract] OR "Macrosomia, Fetal"[Title/Abstract] OR "Perinatal Mortality"[Title/Abstract] OR "Mortalities, Perinatal"[Title/Abstract] OR "Mortality, Perinatal"[Title/Abstract] OR "Perinatal Mortalities"[Title/Abstract] OR "Perinatal Death"[Title/Abstract] OR "Death, Perinatal"[Title/Abstract] OR "Deaths, Perinatal"[Title/Abstract] OR "Perinatal Deaths"[Title/Abstract] OR "Neonatal Death"[Title/Abstract] OR "Death, Neonatal"[Title/Abstract] OR "Deaths, Neonatal"[Title/Abstract] OR "Neonatal Deaths"[Title/Abstract] OR "Congenital Abnormalities"[Title/Abstract] OR "Abnormality, Congenital"[Title/Abstract] OR "Congenital Abnormality"[Title/Abstract] OR Deformities[Title/Abstract] OR Deformity[Title/Abstract] OR "Congenital Defects"[Title/Abstract] OR "Congenital Defect"[Title/Abstract] OR "Defect, Congenital"[Title/Abstract] OR "Defects, Congenital"[Title/Abstract] OR "Abnormalities, Congenital"[Title/Abstract] OR "Birth Defects"[Title/Abstract] OR "Birth Defect"[Title/Abstract] OR "Defect, Birth"[Title/Abstract] OR "Fetal Malformations"[Title/Abstract] OR "Fetal Malformation"[Title/Abstract] OR "Malformation, Fetal"[Title/Abstract] OR "Fetal Anomalies"[Title/Abstract] OR "Anomaly, Fetal"[Title/Abstract] OR "Fetal Anomaly"[Title/Abstract]
