# Supplementary material for: Effect of hydroxychloroquine on pregnancy outcome in patients with SLE: a systematic review and meta-analysis
Source: Lupus Sci Med. 2024 Oct 30;11(2):e001239. doi: 10.1136/lupus-2024-001239 (PMC11529578; doi:10.1136/lupus-2024-001239)
Supplement: online supplemental file 2 [file lupus-11-2-s002.pdf]

Supplementary material B: Quality assessment of each included study

No.	Studies	Year	Selection	Comparability	Outcome	Total
1	Abd et al.	2020	4	2	1	7
2	Canti et al.	2021	4	2	2	8
3	Clowse et al.	2006	4	2	2	8
4	Do et al.	2020	4	2	1	7
5	Kroese et al.	2017	4	0	1	5
6	Leroux et al.	2015	4	2	1	7
7	Seo et al.	2019	4	2	1	7
8	Cao ZJ et al.	2012	4	0	1	5
9	Chen GL.	2021	4	1	3	8
10	Deng RR.	2017	4	0	3	7
11	Diao MX.	2016	4	1	1	6
12	Wu FQ et al.	2019	4	1	1	6
13	Lin QR et al.	2021	4	1	1	6
14	Liu XY et al.	2015	4	1	1	6
15	Liu YQ et al.	2013	4	1	1	6
16	Zheng CS et al.	2015	4	1	1	6
17	Wang LH et al.	2018	4	1	1	6
18	Wang WJ et al.	2018	4	1	1	6
19	Wu MY.	2014	4	1	3	8
20	Zhang HQ.	2019	4	1	1	6
21	Zhou L et al.	2017	4	0	3	7
