# Supplementary material for: Effect of hydroxychloroquine on pregnancy outcome in patients with SLE: a systematic review and meta-analysis
Source: Lupus Sci Med. 2024 Oct 30;11(2):e001239. doi: 10.1136/lupus-2024-001239 (PMC11529578; doi:10.1136/lupus-2024-001239)
Supplement: online supplemental file 3 [file lupus-11-2-s003.pdf]

Supplementary material C: Sensitivity analysis

C.1. Flare

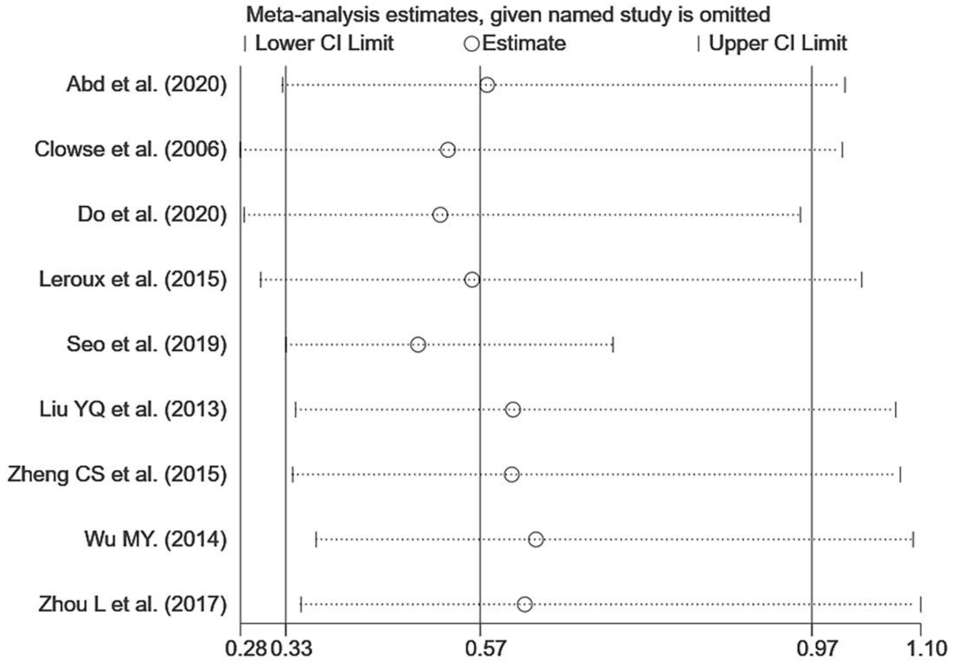

C.2. SLEDAI:first trimester

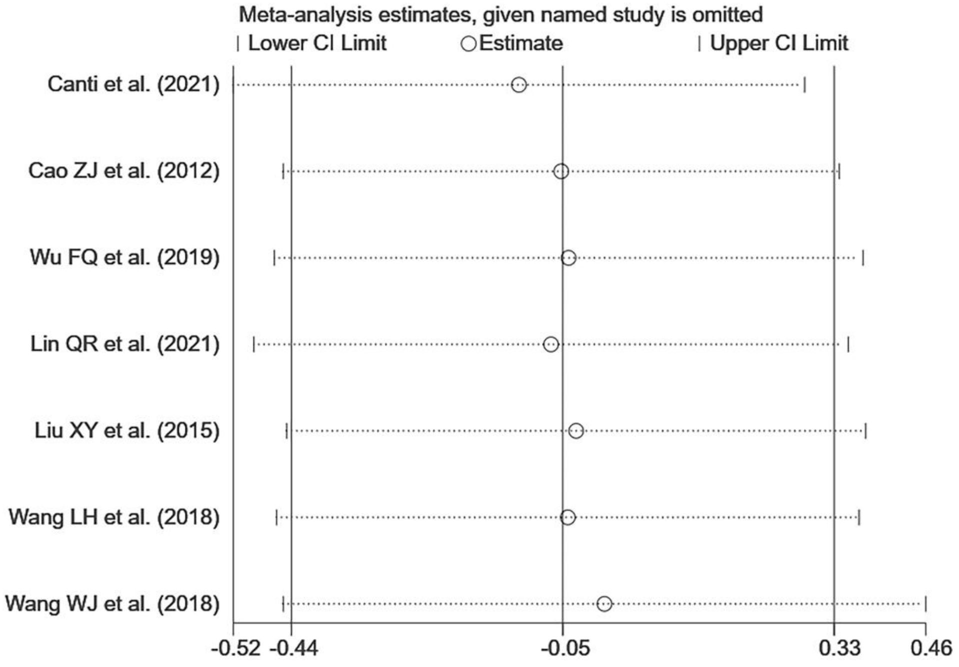

C.3. SLEDAI:second trimester

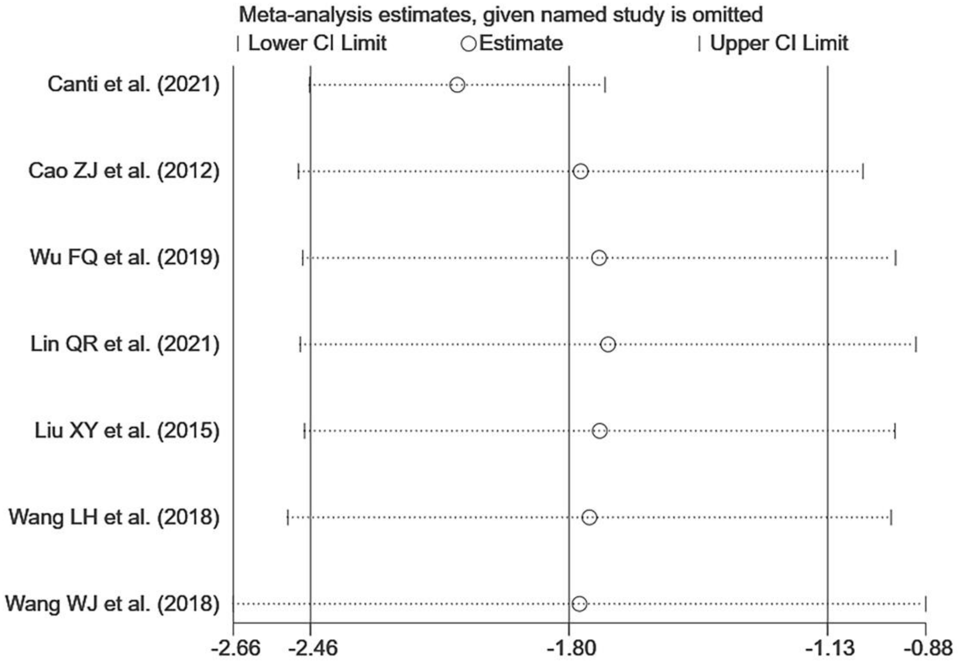

C.4. SLEDAI:third trimester

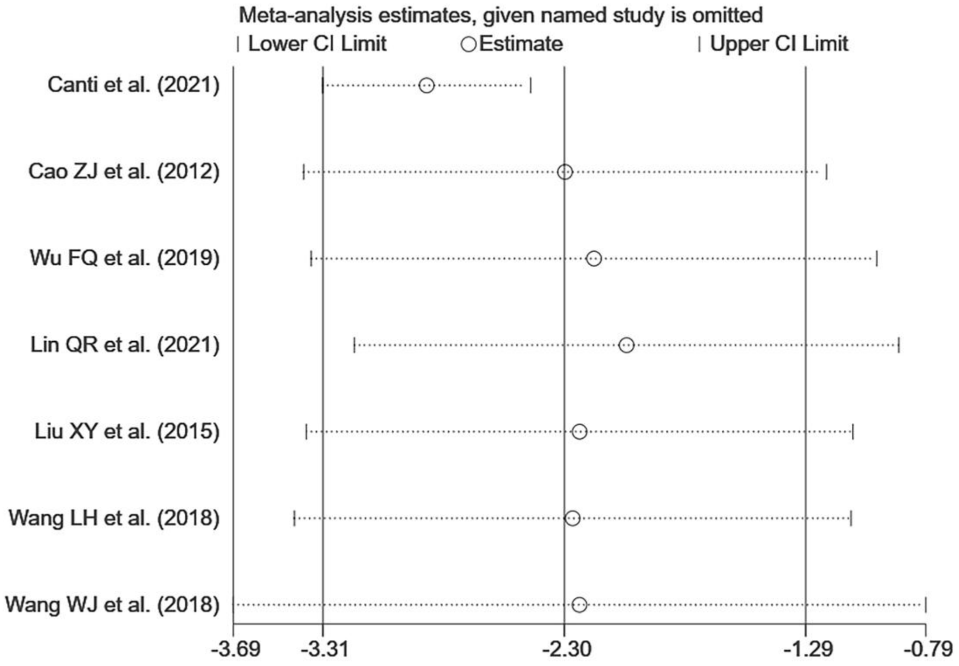

C.5. Full-term birth

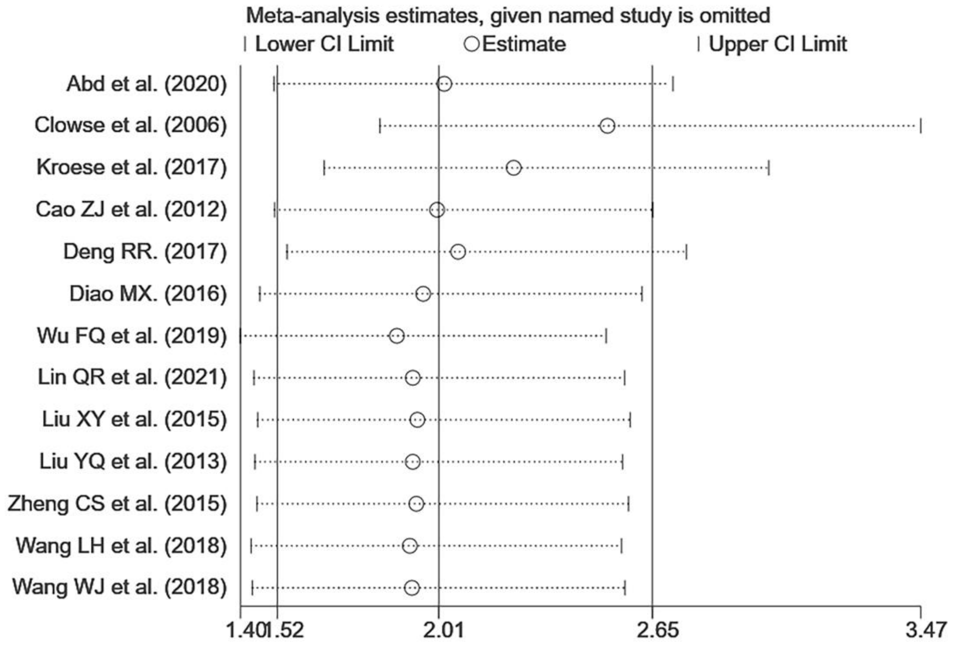

C.6. Preterm birth

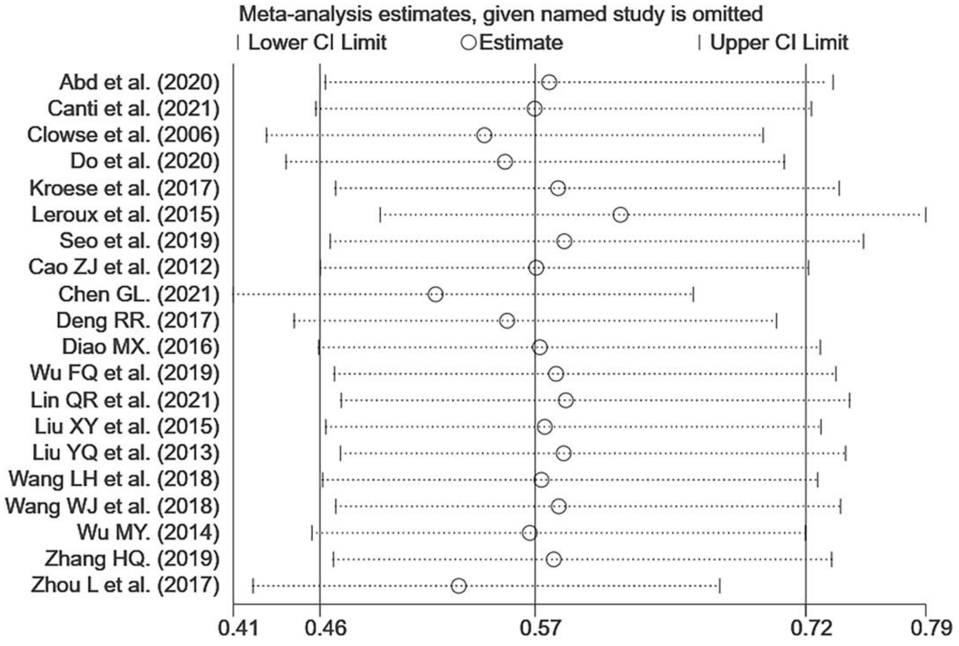

C.7. Miscarriage

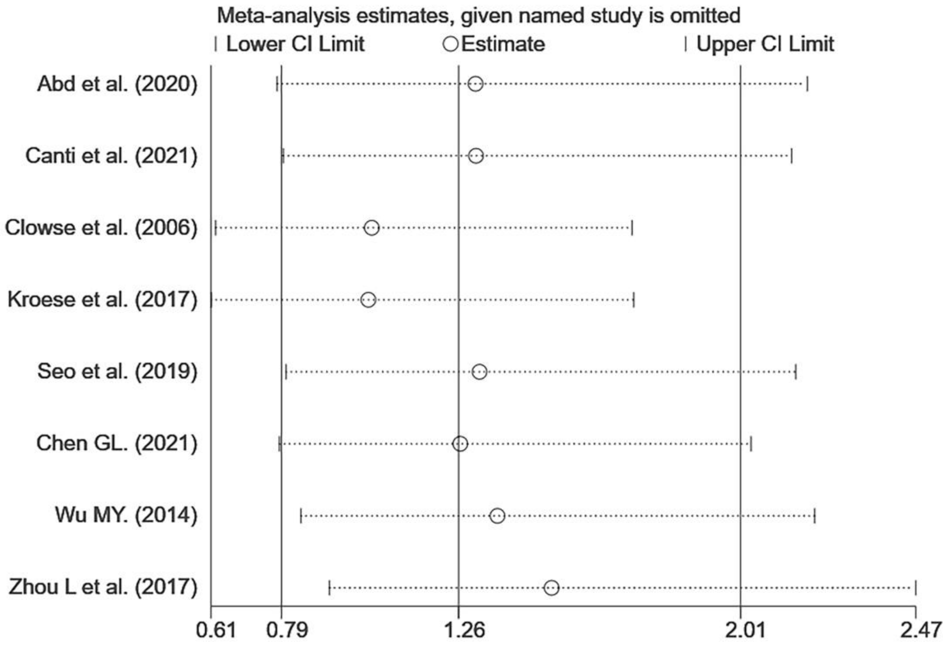

C.8. Stillbirth

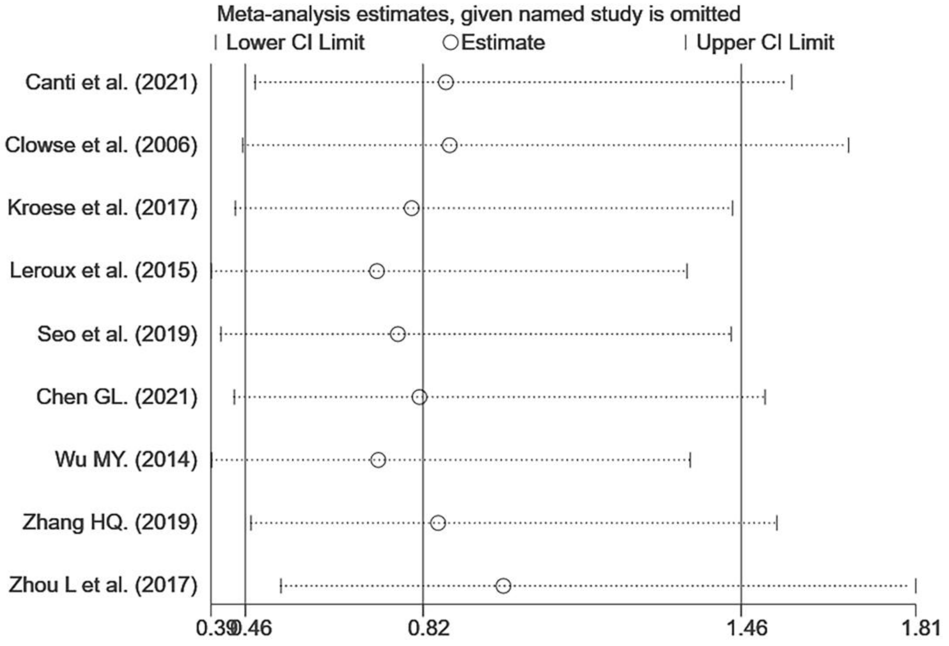

C.9. Fetal distress

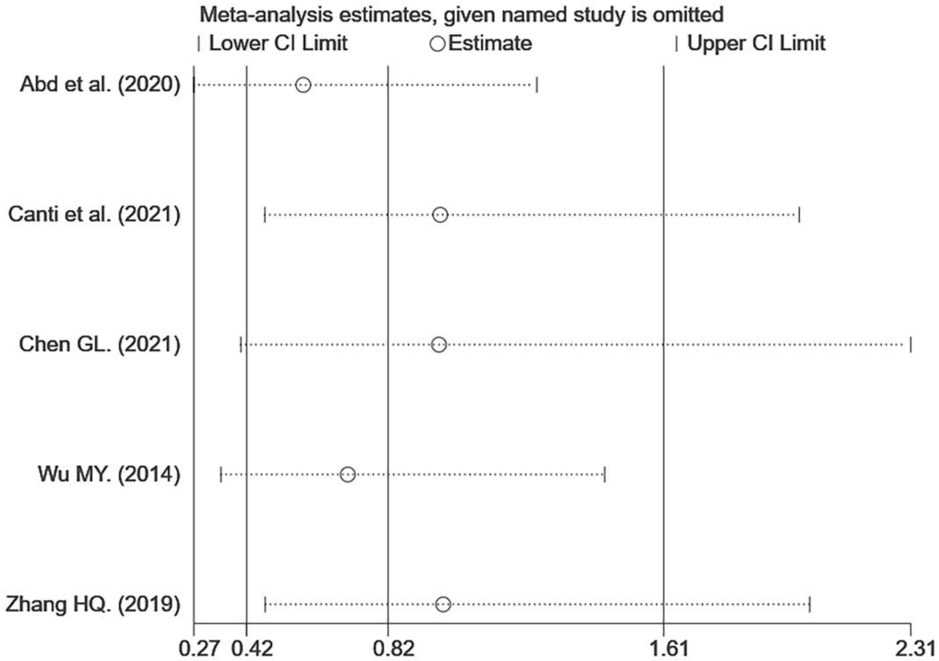

C.10. IUGR

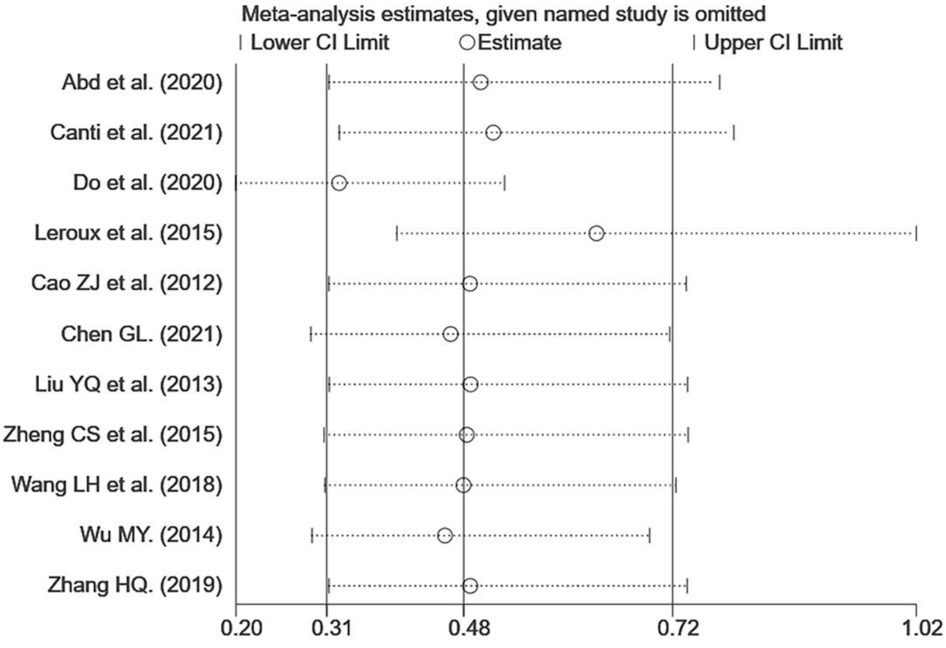

C.11. Low birth weight

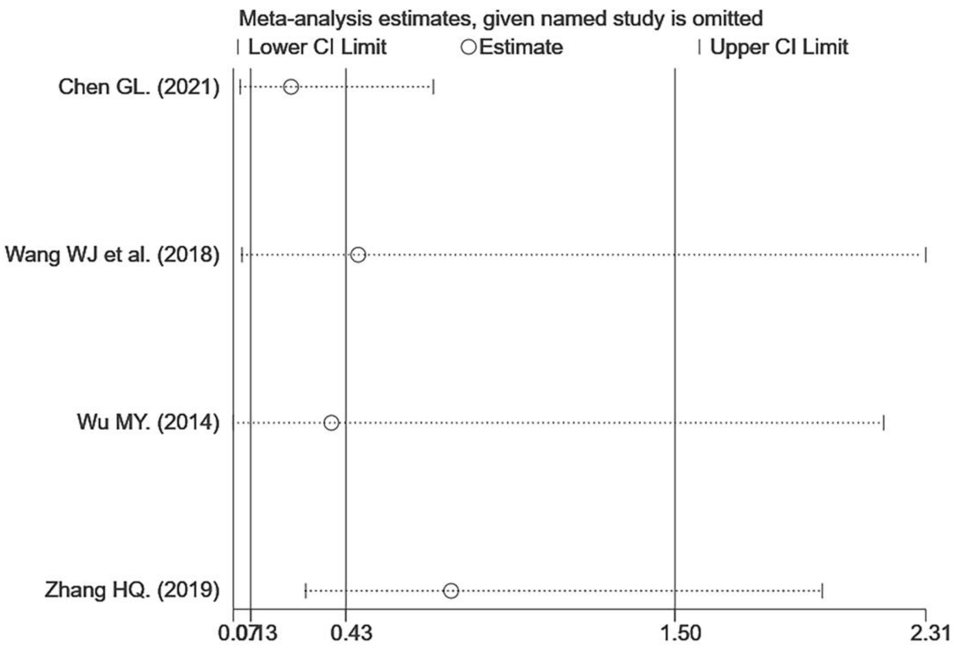

C.12. SGA

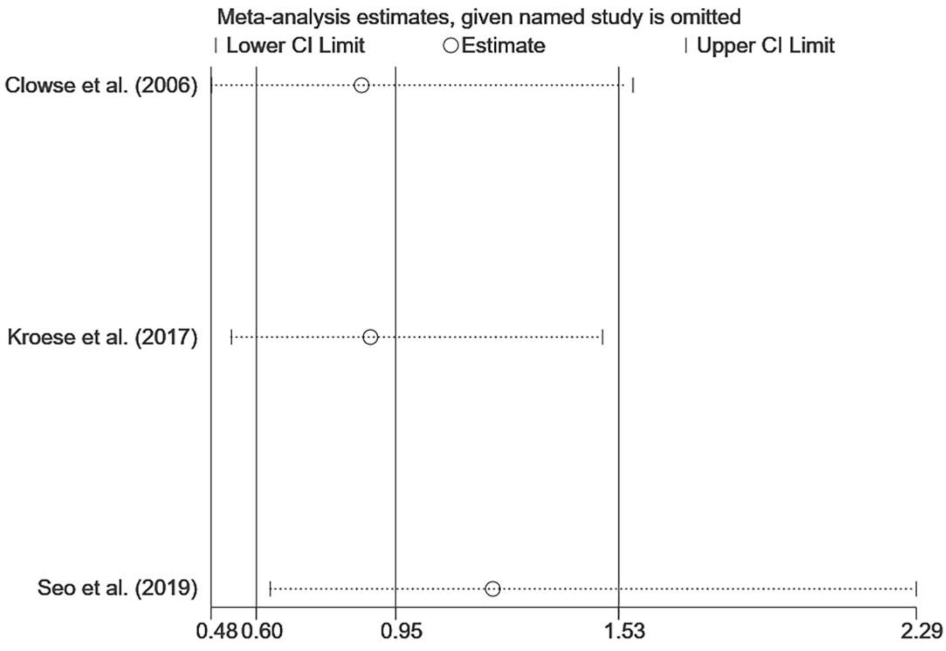

C.13. Gestational hypertension

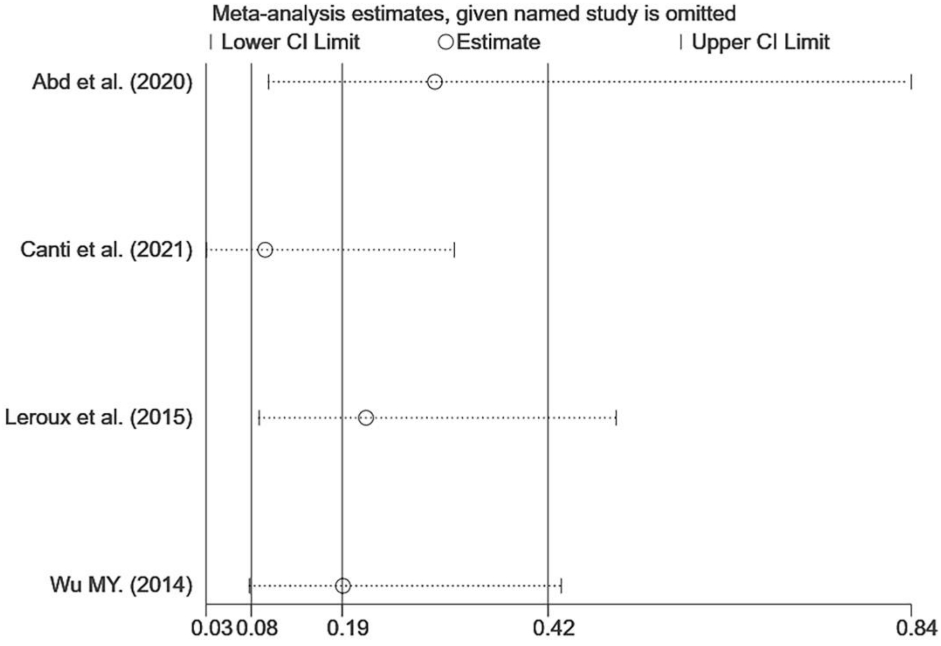

C.14. Pre-eclampsia

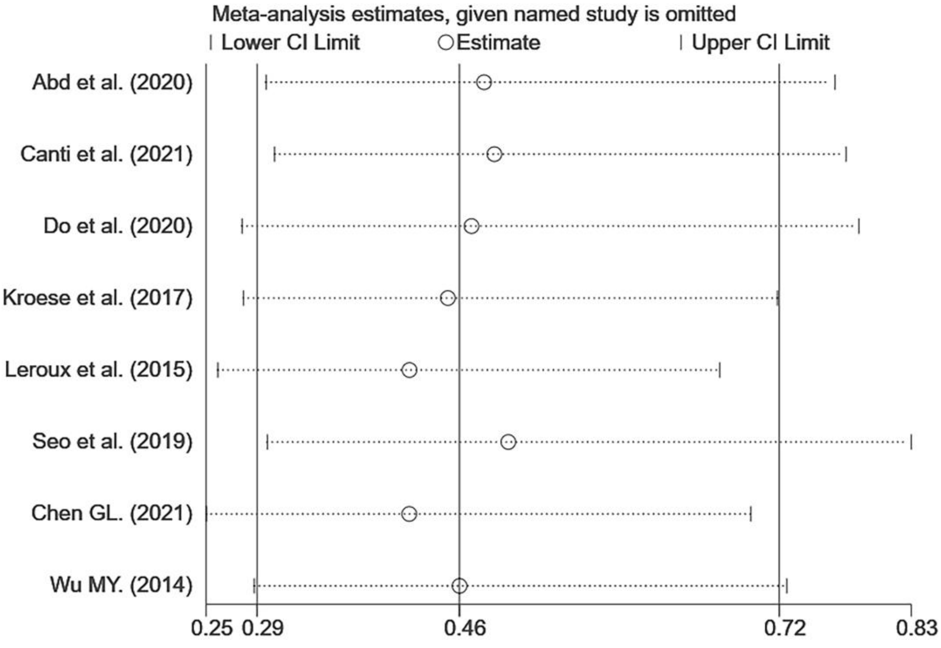

C.15. Gestational diabetes mellitus

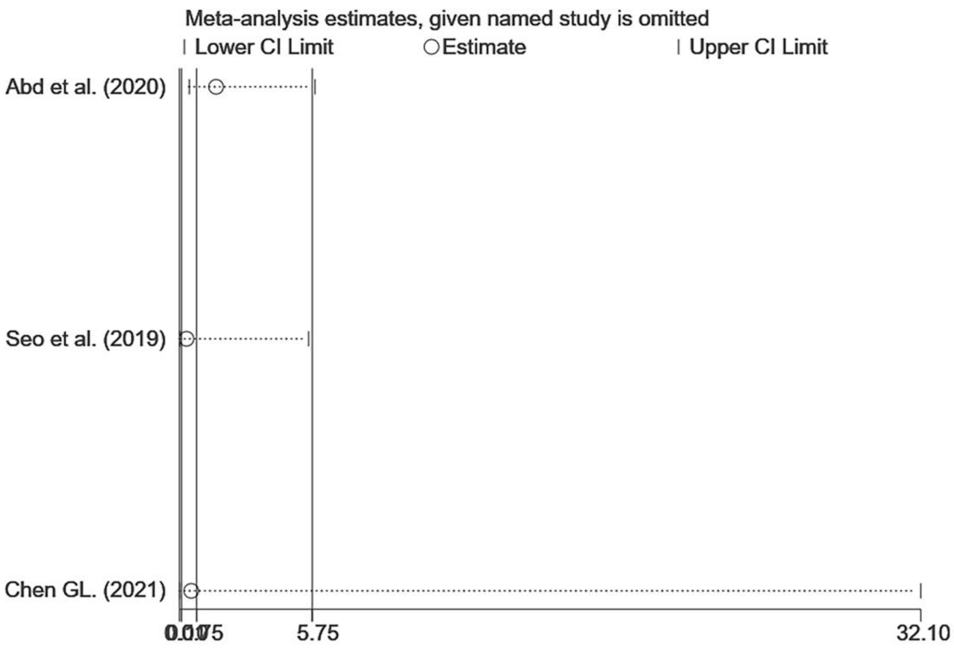
Meta-analysis estimates, given named study is omitted
Lower CI Limit
Estimate
Upper CI Limit
Abd et al. (2020)
Seo et al. (2019)
Chen GL. (2021)
5.75
32.10
