# Supplementary material for: Effect of hydroxychloroquine on pregnancy outcome in patients with SLE: a systematic review and meta-analysis
Source: Lupus Sci Med. 2024 Oct 30;11(2):e001239. doi: 10.1136/lupus-2024-001239 (PMC11529578; doi:10.1136/lupus-2024-001239)
Supplement: online supplemental file 4 [file lupus-11-2-s004.pdf]

Supplementary material D: Analysis of Publication Bias

D.1. Funnel plot of flare risk

The funnel plot shows no evidence of publication bias. Egger's test for a regression intercept gave a p-value of 0.234, indicating no evidence of publication bias.

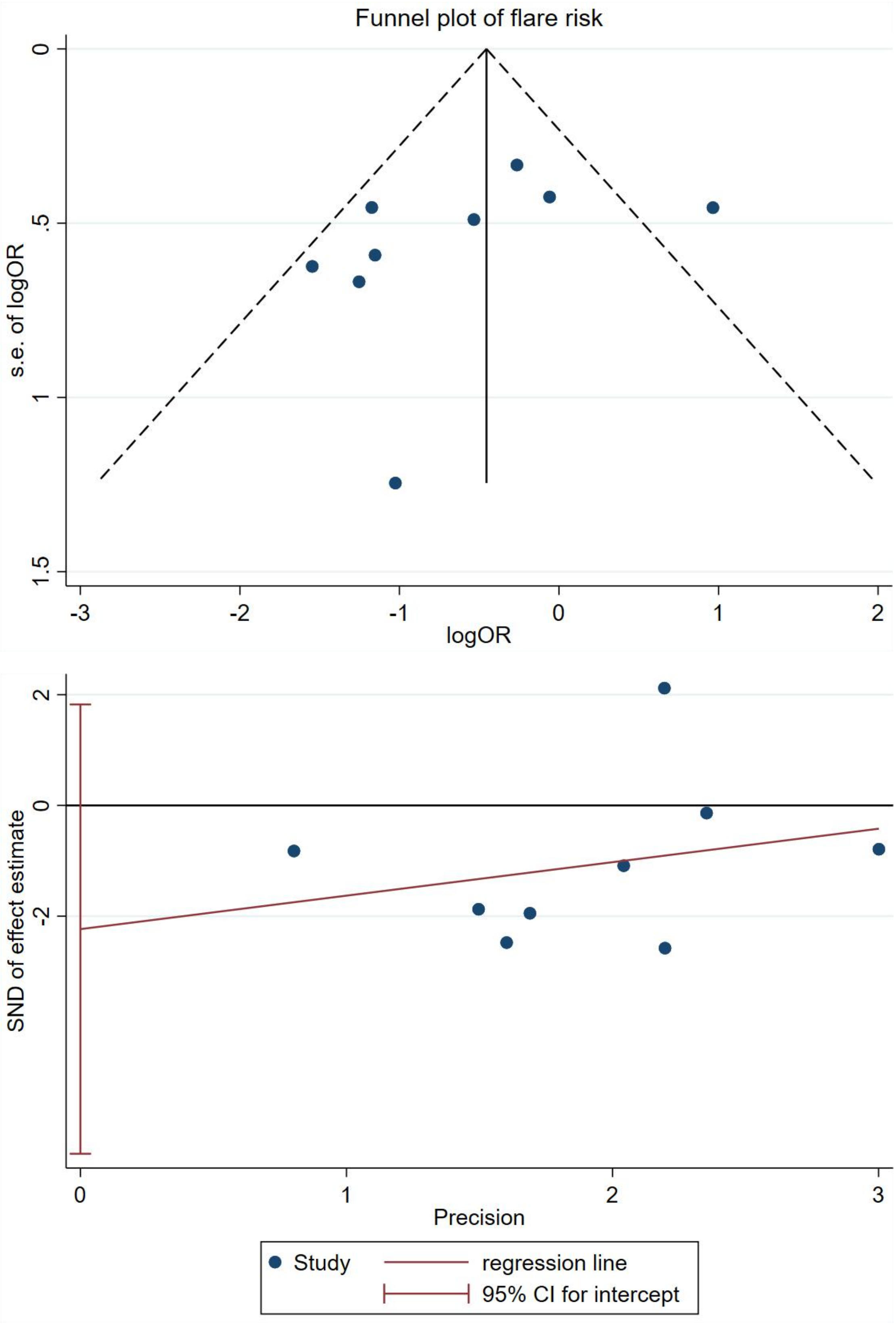

D.2. Funnel plot of the first trimester of SLEDAI risk

The funnel plot shows no evidence of publication bias. Egger's test for a regression intercept gave a p-value of 0.282, indicating no evidence of publication bias.

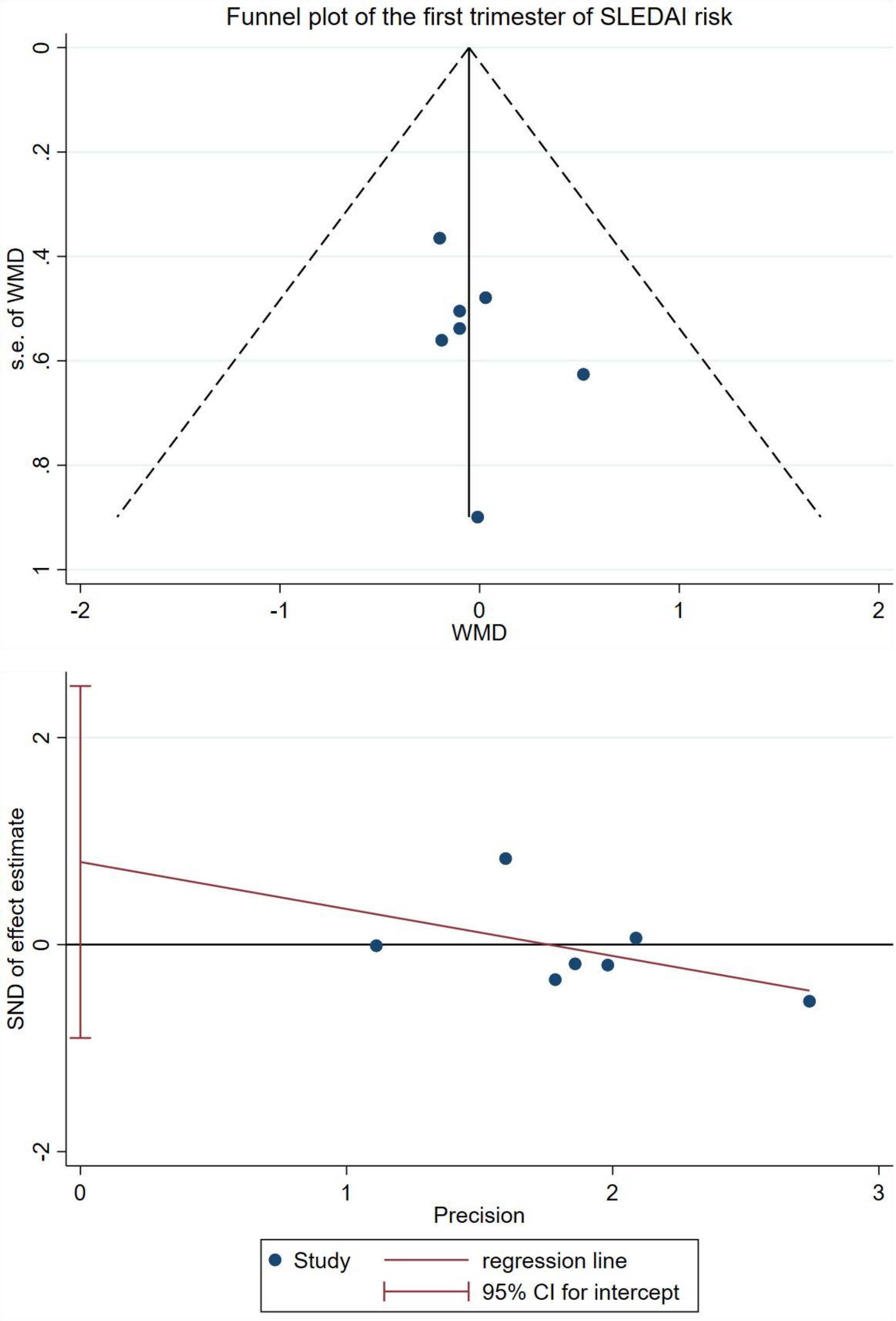

D.3. Funnel plot of the second trimester of SLEDAI risk

The funnel plot shows no evidence of publication bias. Egger's test for a regression intercept gave a p-value of 0.826, indicating no evidence of publication bias.

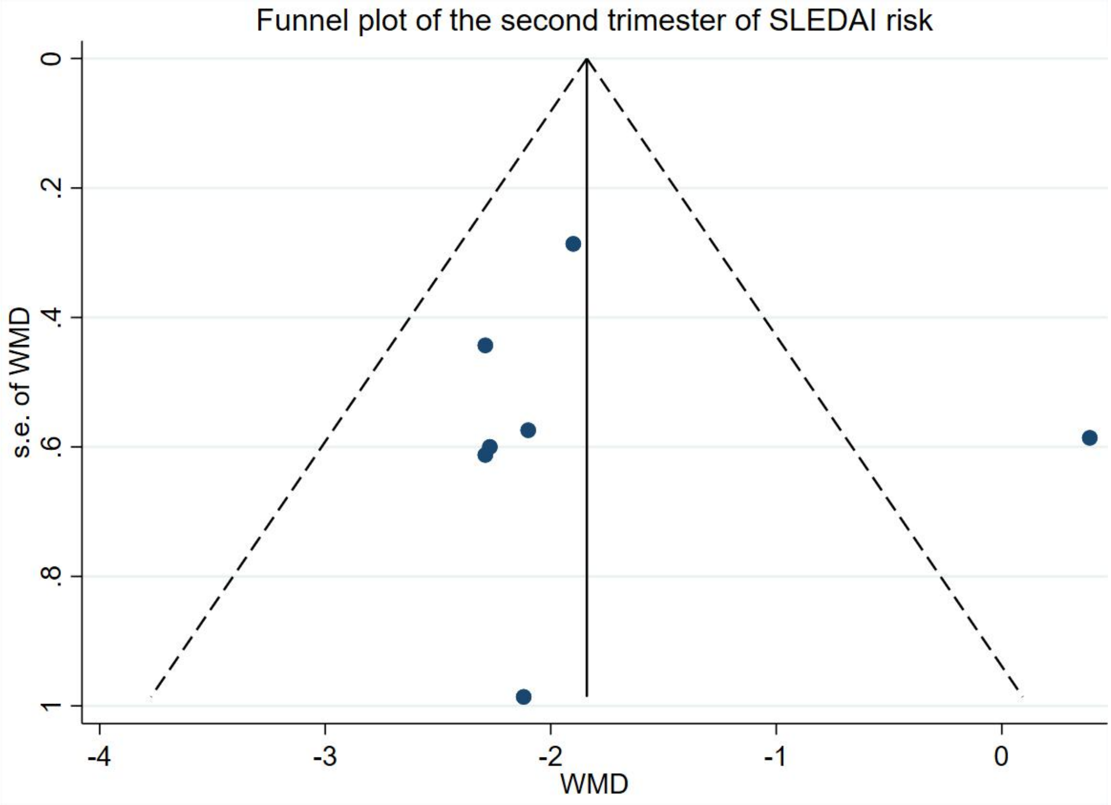

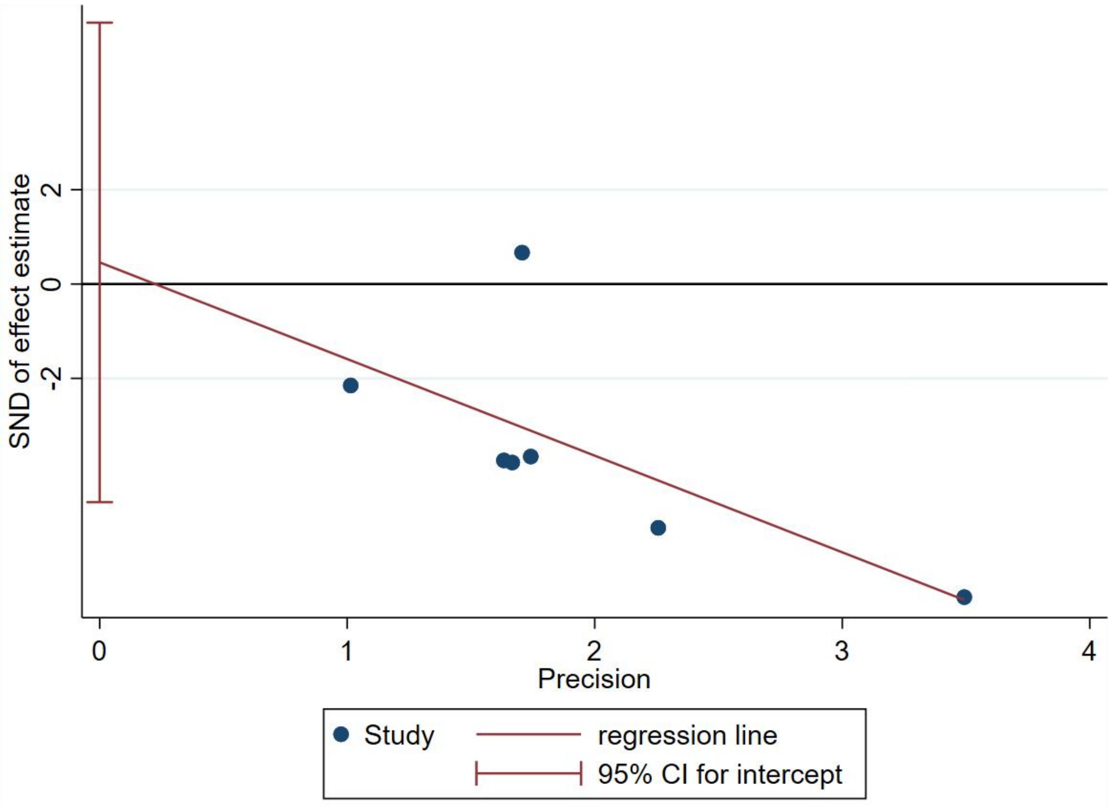

D.4. Funnel plot of the third trimester of SLEDAI risk

The funnel plot shows no evidence of publication bias. Egger's test for a regression intercept gave

a p-value of 0.666, indicating no evidence of publication bias.

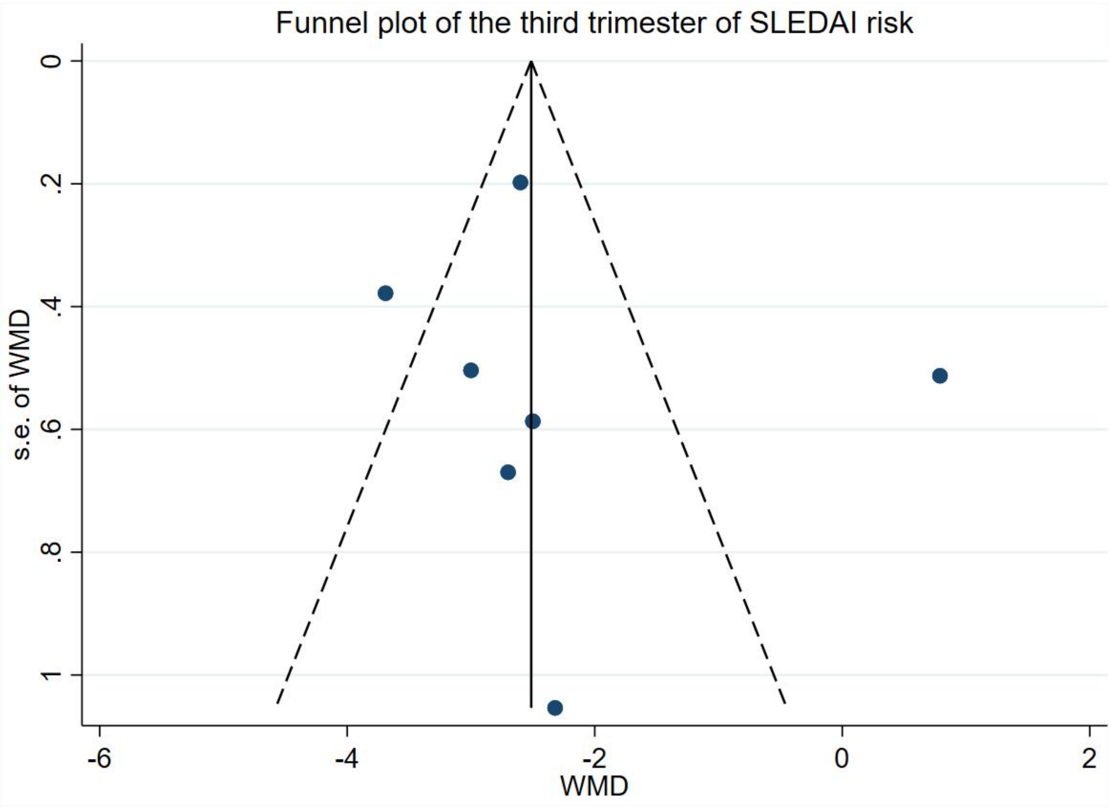

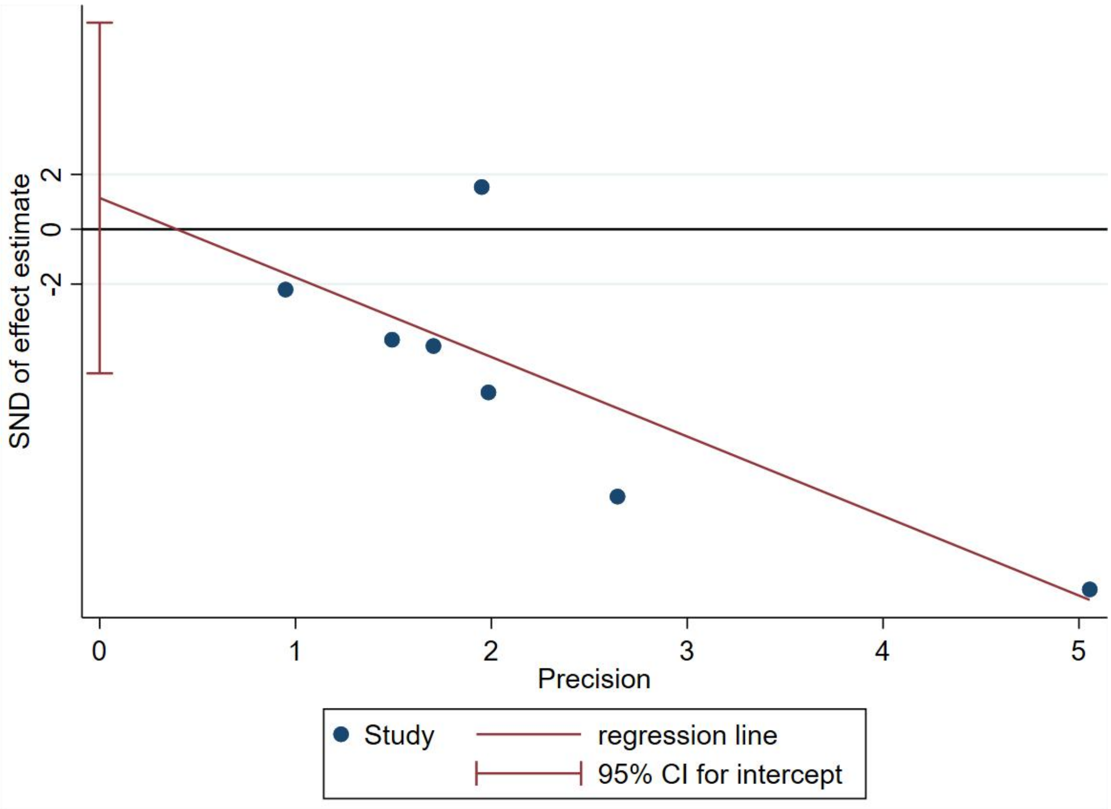

D.5. Funnel plot of full-term birth risk

The funnel plot shows evidence of publication bias. Egger's test for a regression intercept gave a p-value of 0.003, indicating evidence of publication bias.

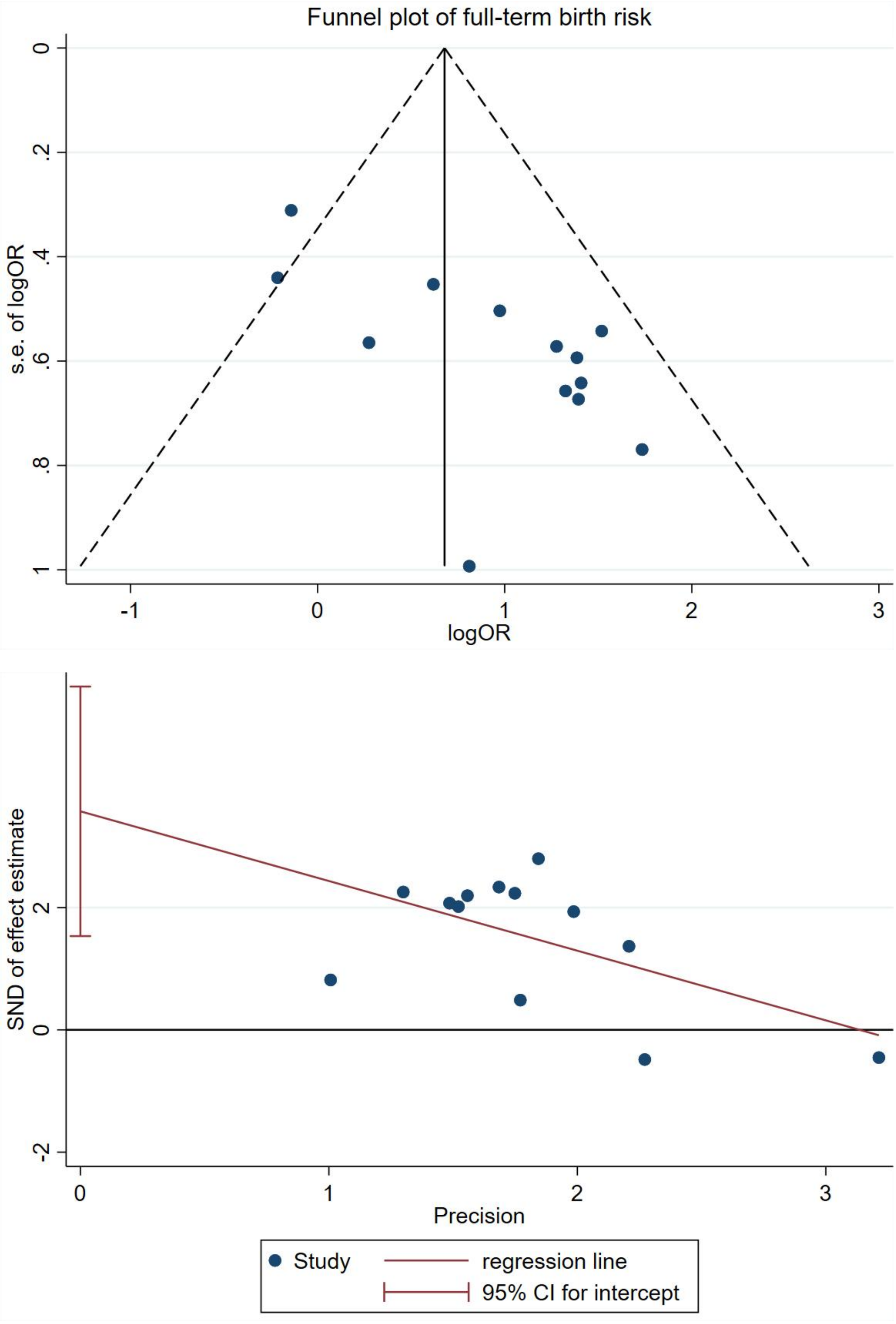

D.6. Funnel plot of preterm birth risk

The funnel plot shows no evidence of publication bias. Egger's test for a regression intercept gave a p-value of 0.167, indicating no evidence of publication bias.

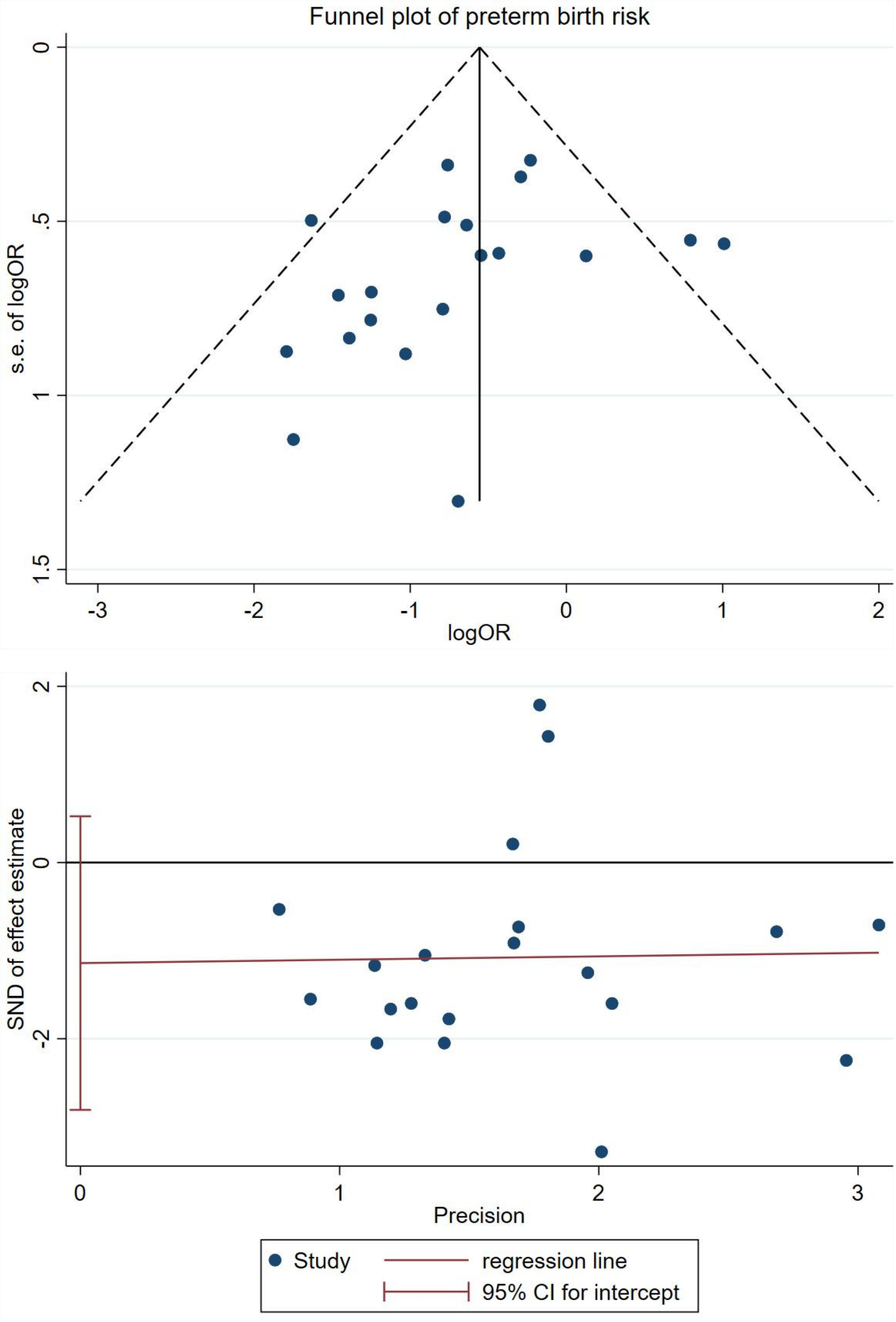

D.7. Funnel plot of miscarriage risk

The funnel plot shows no evidence of publication bias. Egger's test for a regression intercept gave a p-value of 0.068, indicating no evidence of publication bias.

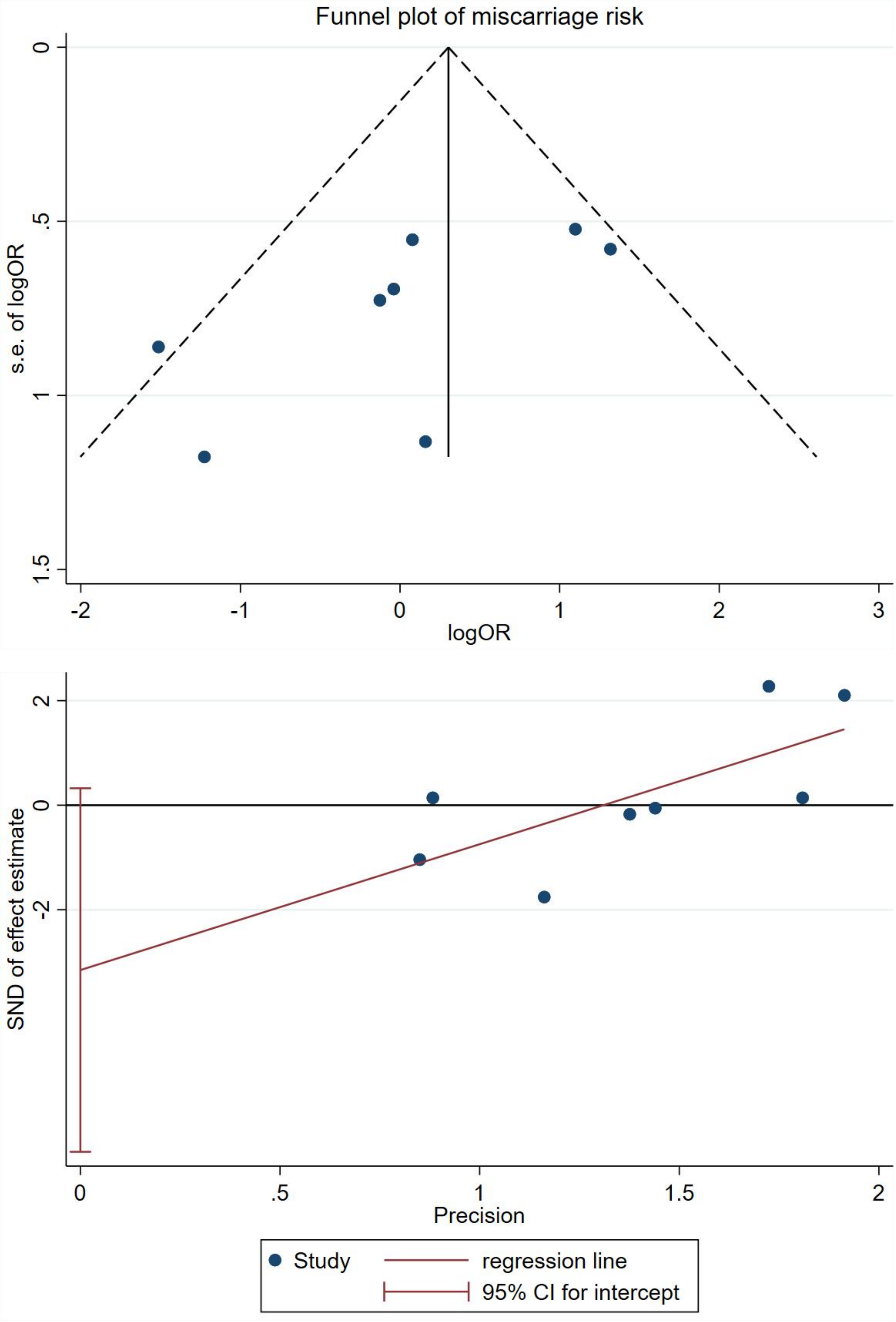

D.8. Funnel plot of stillbirth risk

The funnel plot shows no evidence of publication bias. Egger's test for a regression intercept gave a p-value of 0.245, indicating no evidence of publication bias.

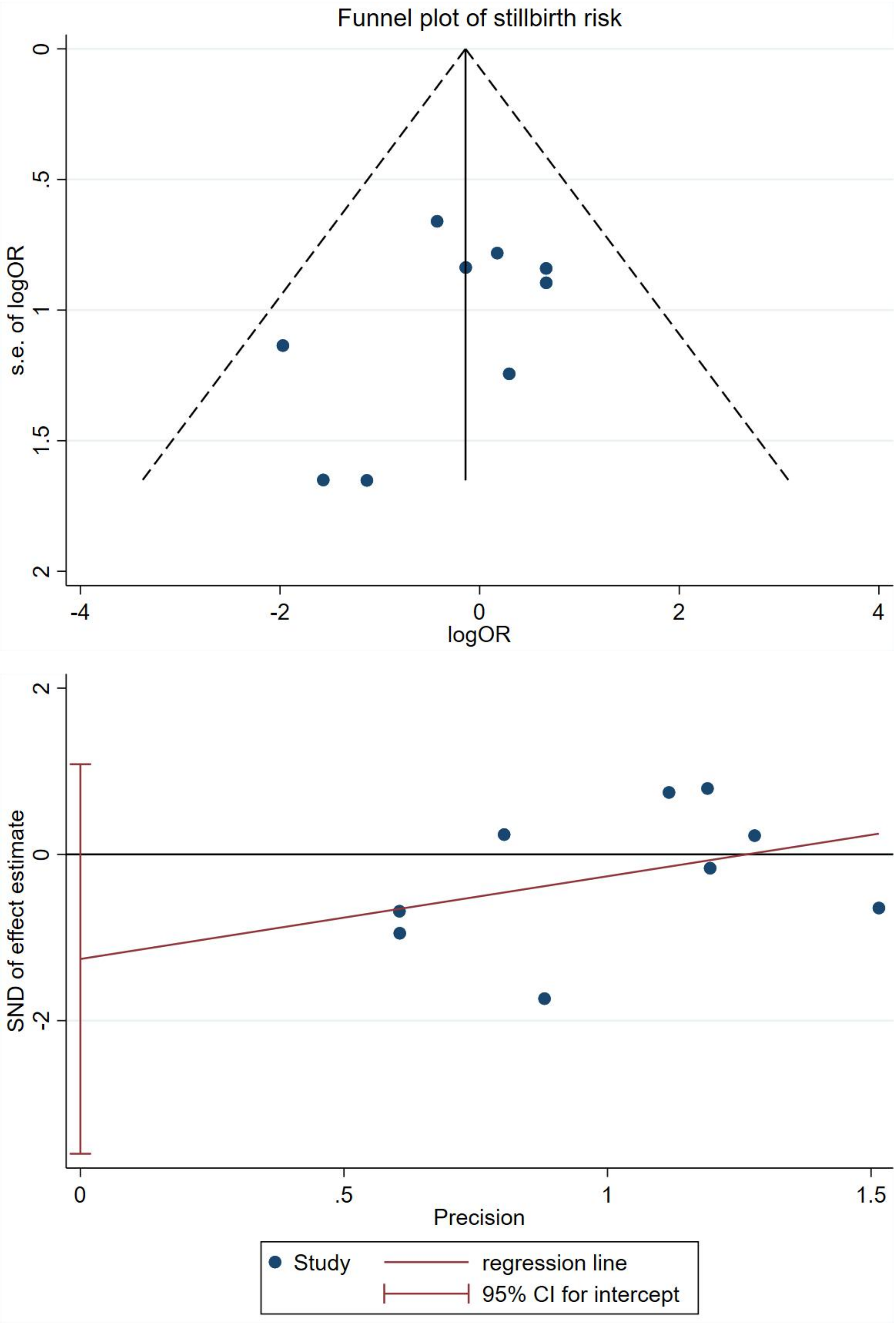

D.9. Funnel plot of fetal distress risk

The funnel plot shows no evidence of publication bias. Egger's test for a regression intercept gave a p-value of 0.846, indicating no evidence of publication bias.

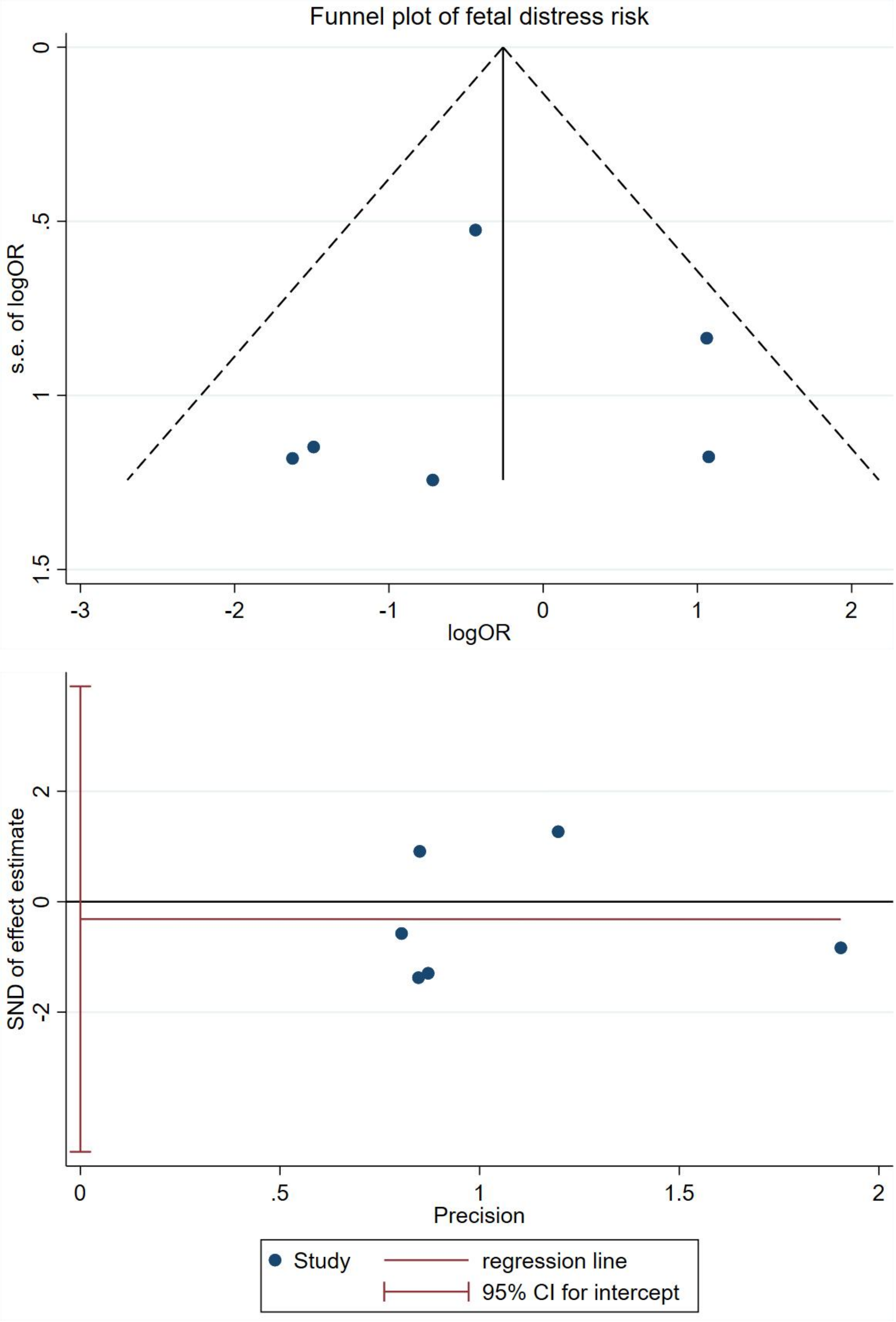

D.10. Funnel plot of IUGR risk

The funnel plot shows no evidence of publication bias. Egger's test for a regression intercept gave a p-value of 0.711, indicating no evidence of publication bias.

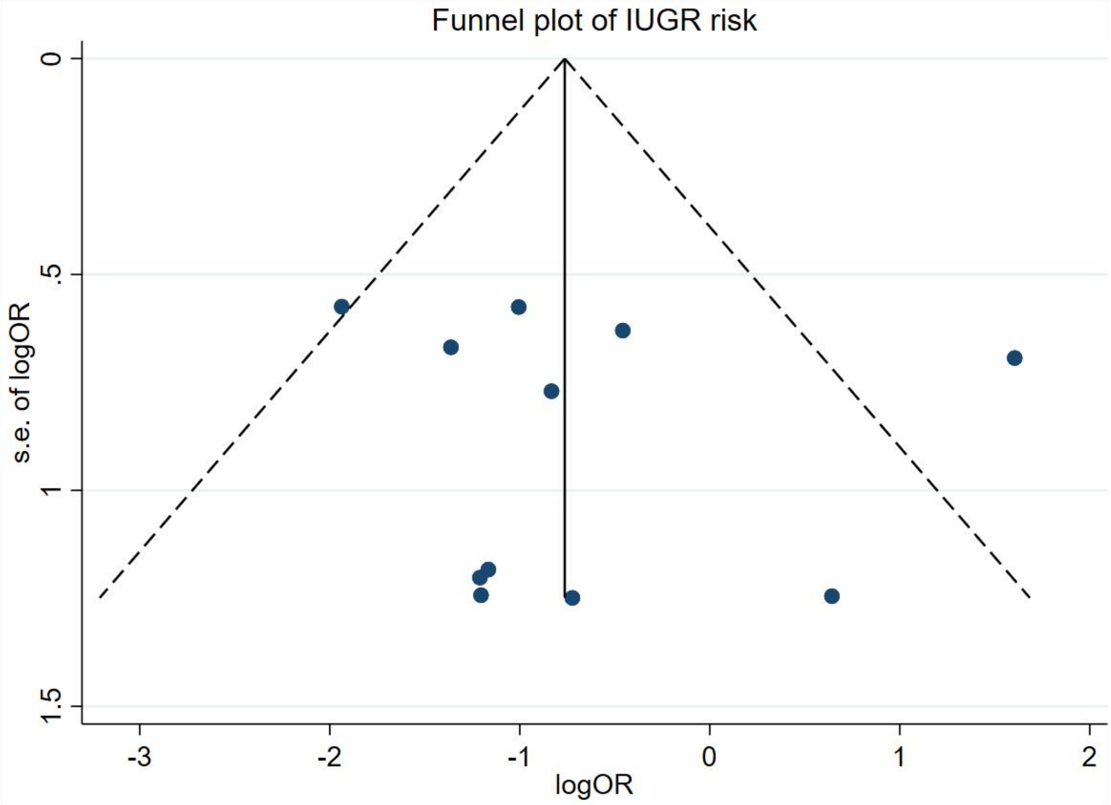

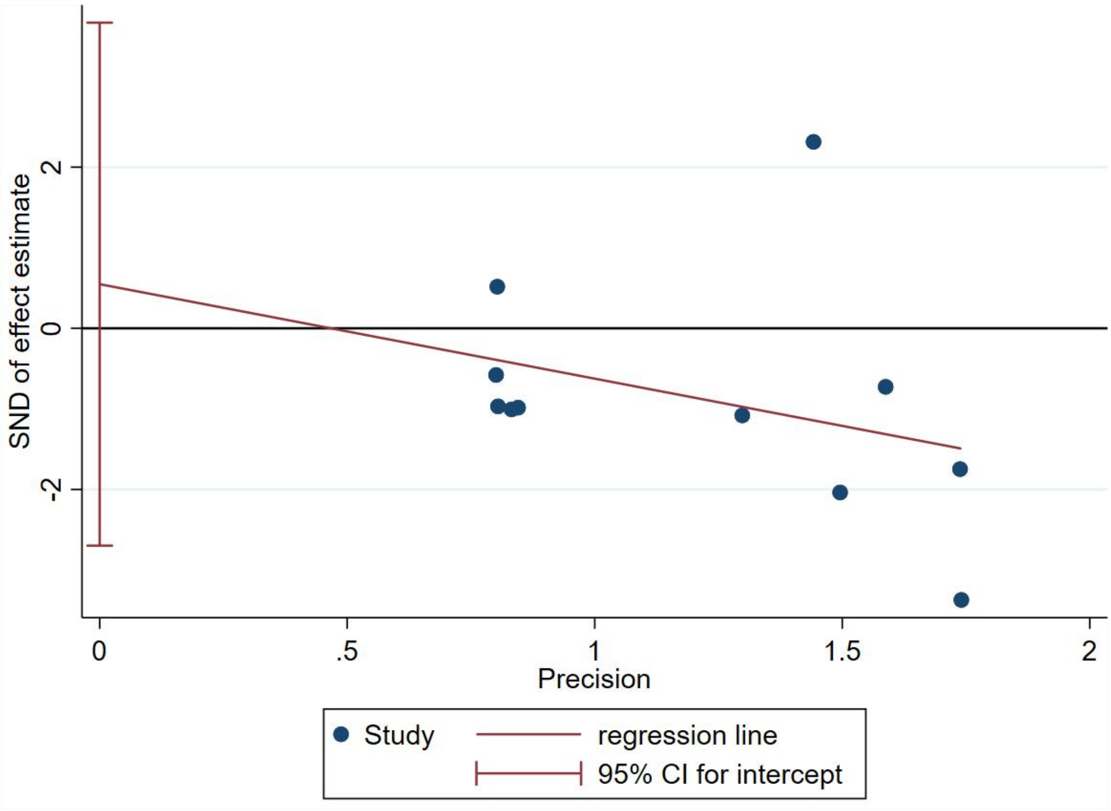

D.11. Funnel plot of low birth weight risk

The funnel plot shows no evidence of publication bias. Egger's test for a regression intercept gave a p-value of 0.477, indicating no evidence of publication bias.

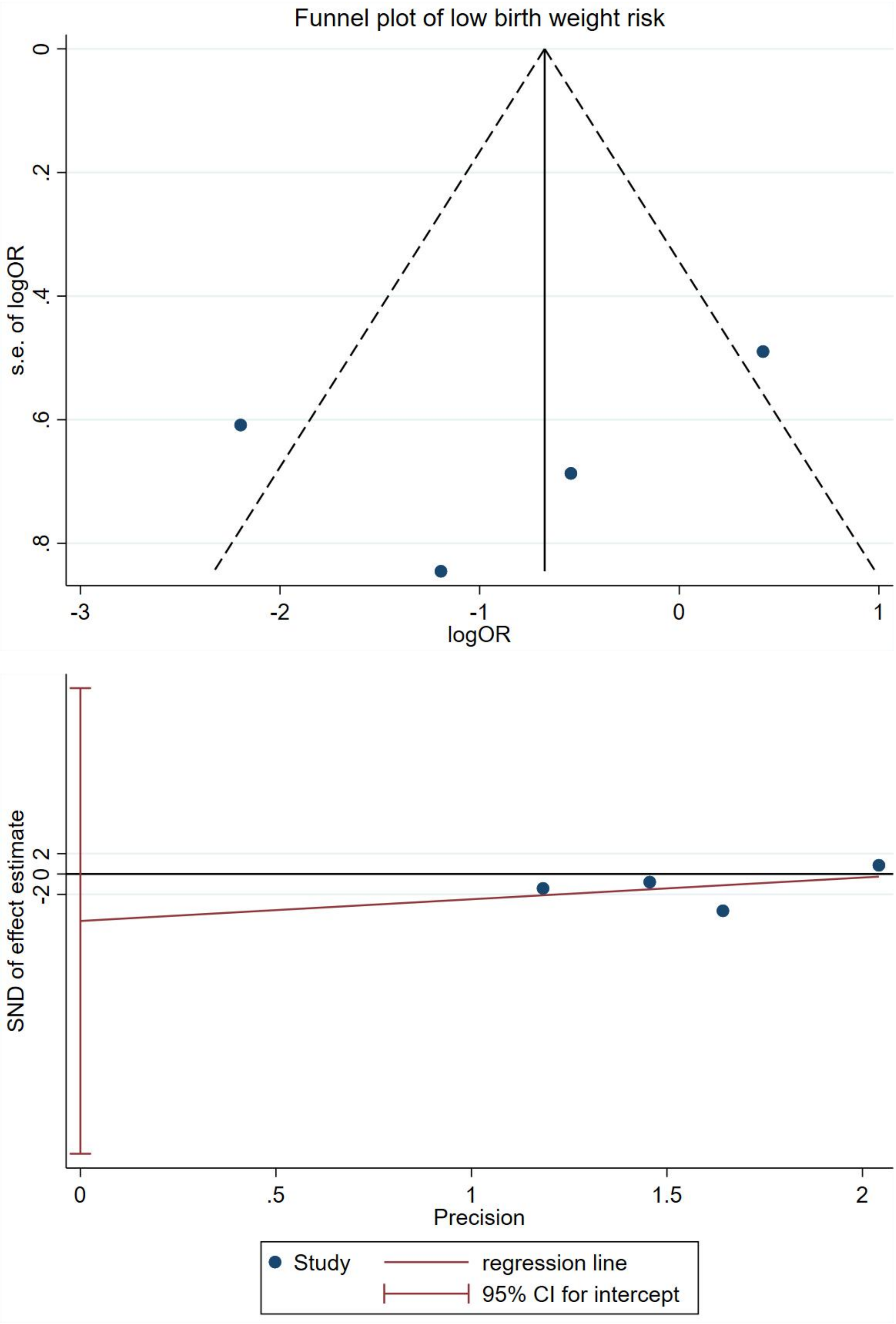

D.12. Funnel plot of SGA risk

The funnel plot shows no evidence of publication bias. Egger's test for a regression intercept gave a p-value of 0.386, indicating no evidence of publication bias.

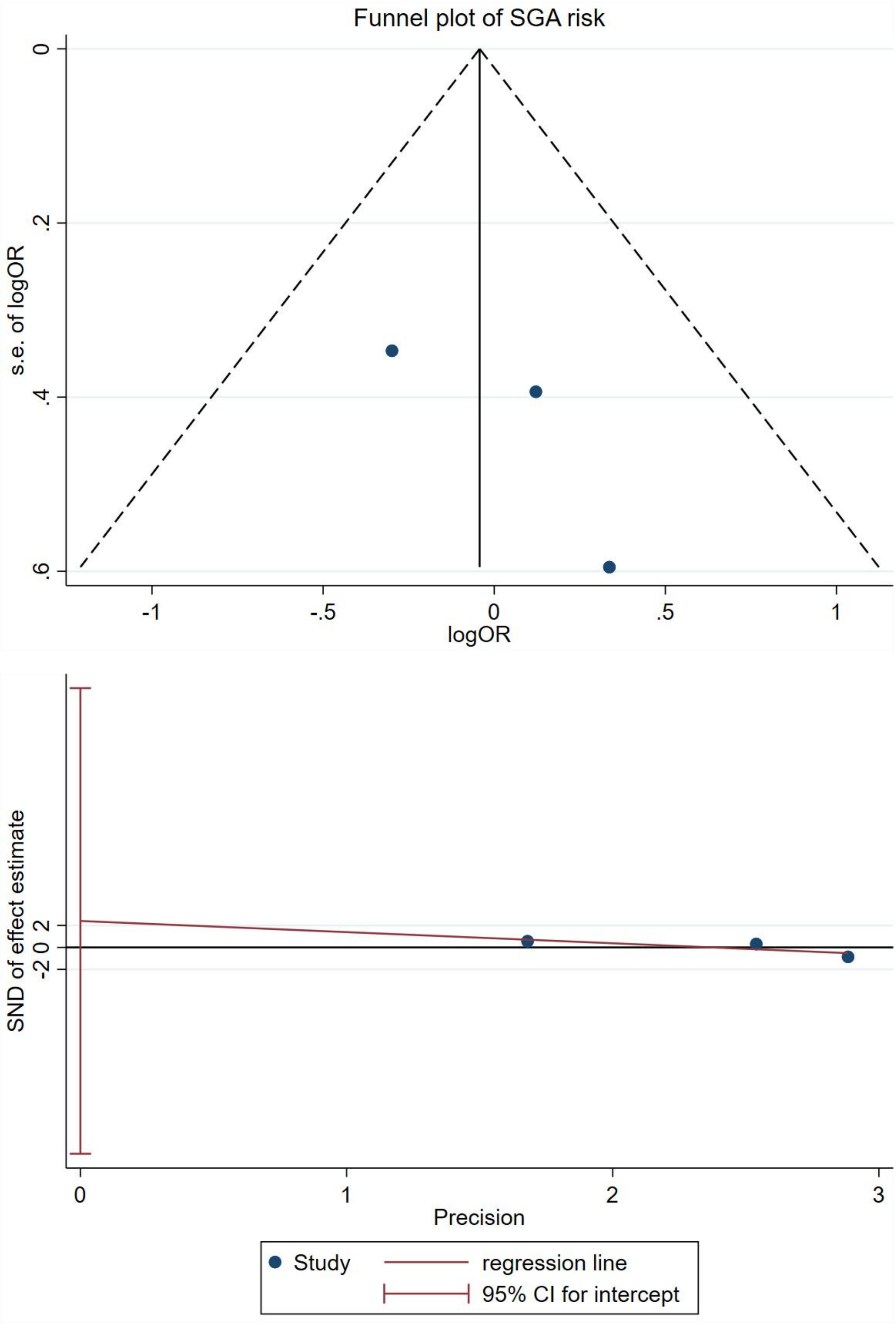

D.13. Funnel plot of gestational hypertension risk

The funnel plot shows no evidence of publication bias. Egger's test for a regression intercept gave a p-value of 0.730, indicating no evidence of publication bias.

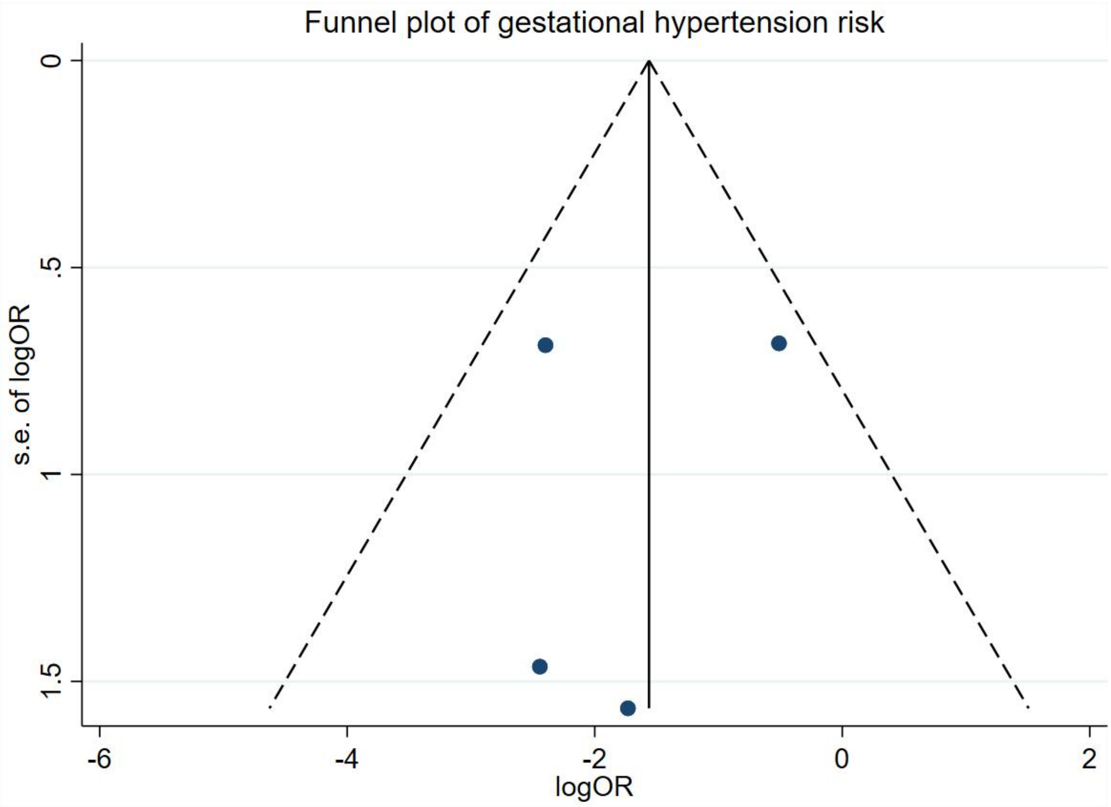

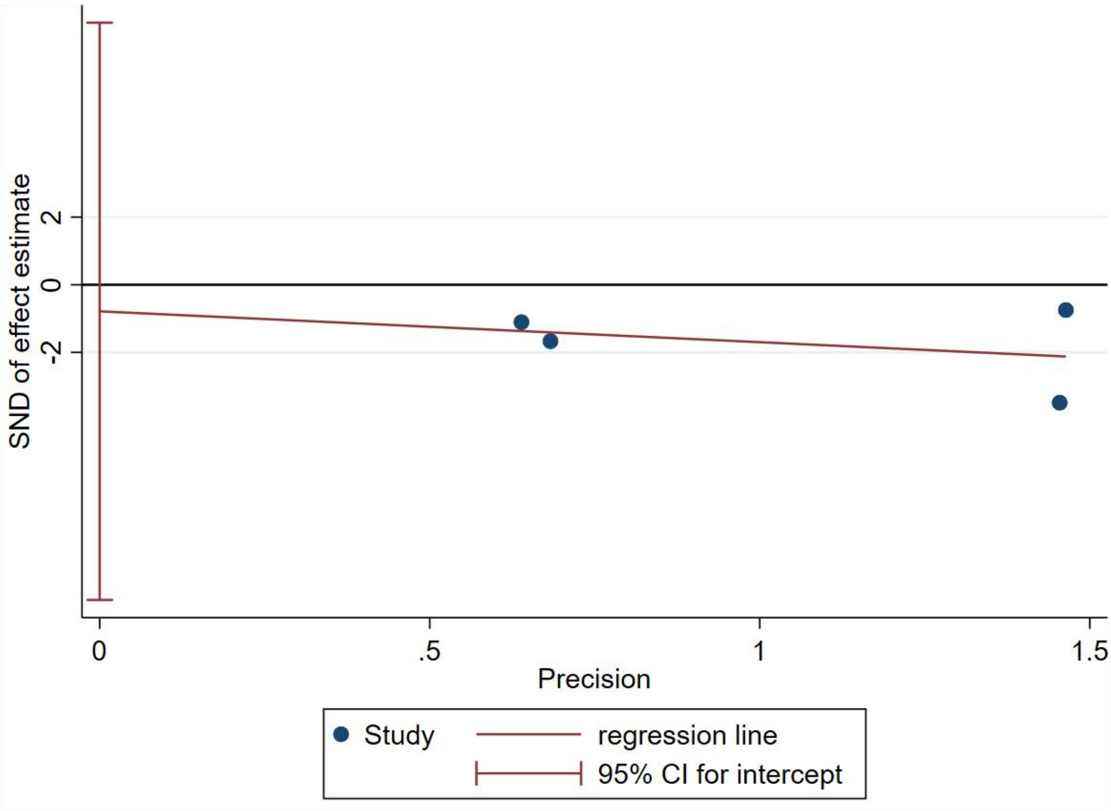

D.14. Funnel plot of pre-eclampsia risk

The funnel plot shows no evidence of publication bias. Egger's test for a regression intercept gave a p-value of 0.622, indicating no evidence of publication bias.

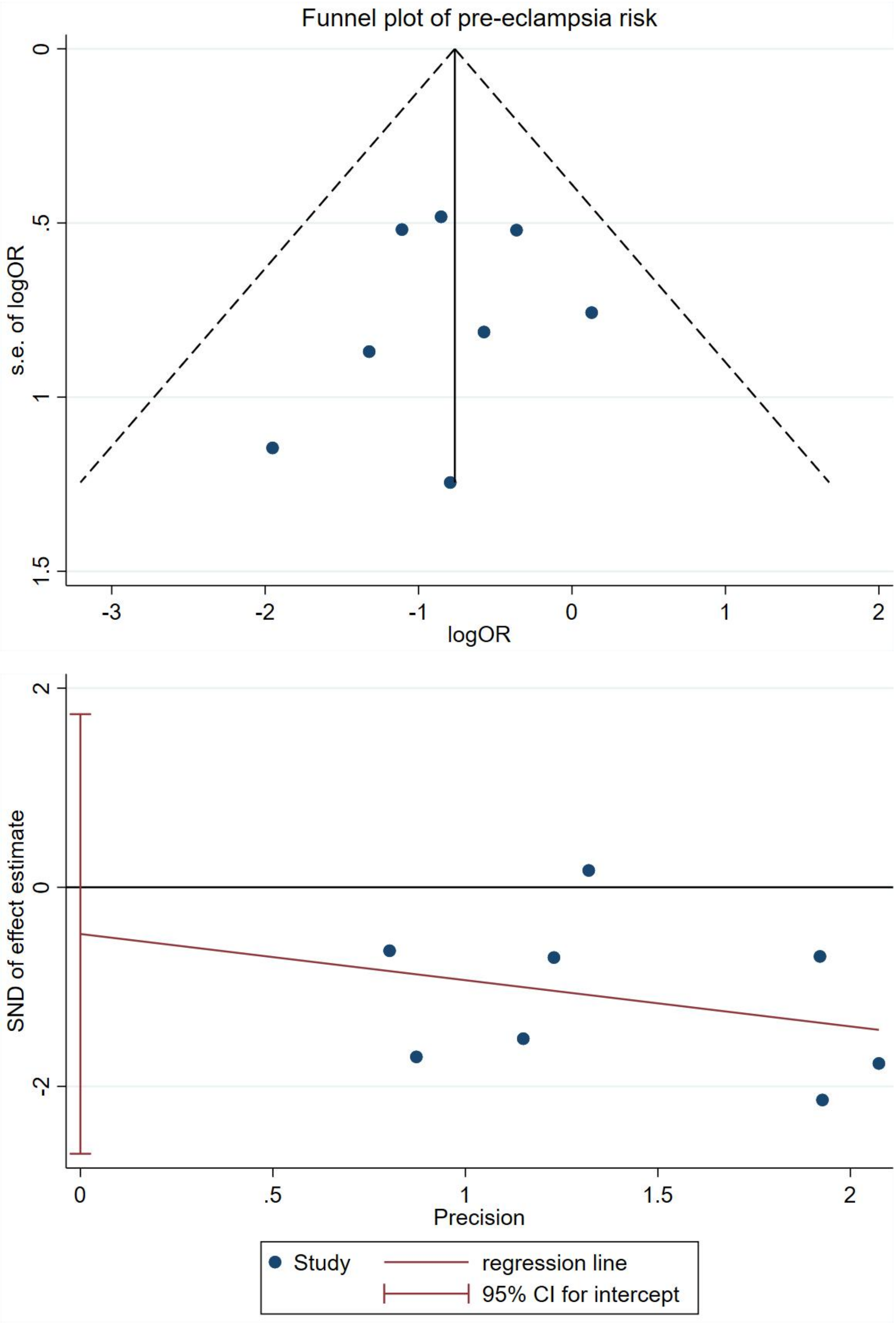

D.15. Funnel plot of gestational diabetes mellitus risk

The funnel plot shows no evidence of publication bias. Egger's test for a regression intercept gave a p-value of 0.632, indicating no evidence of publication bias.

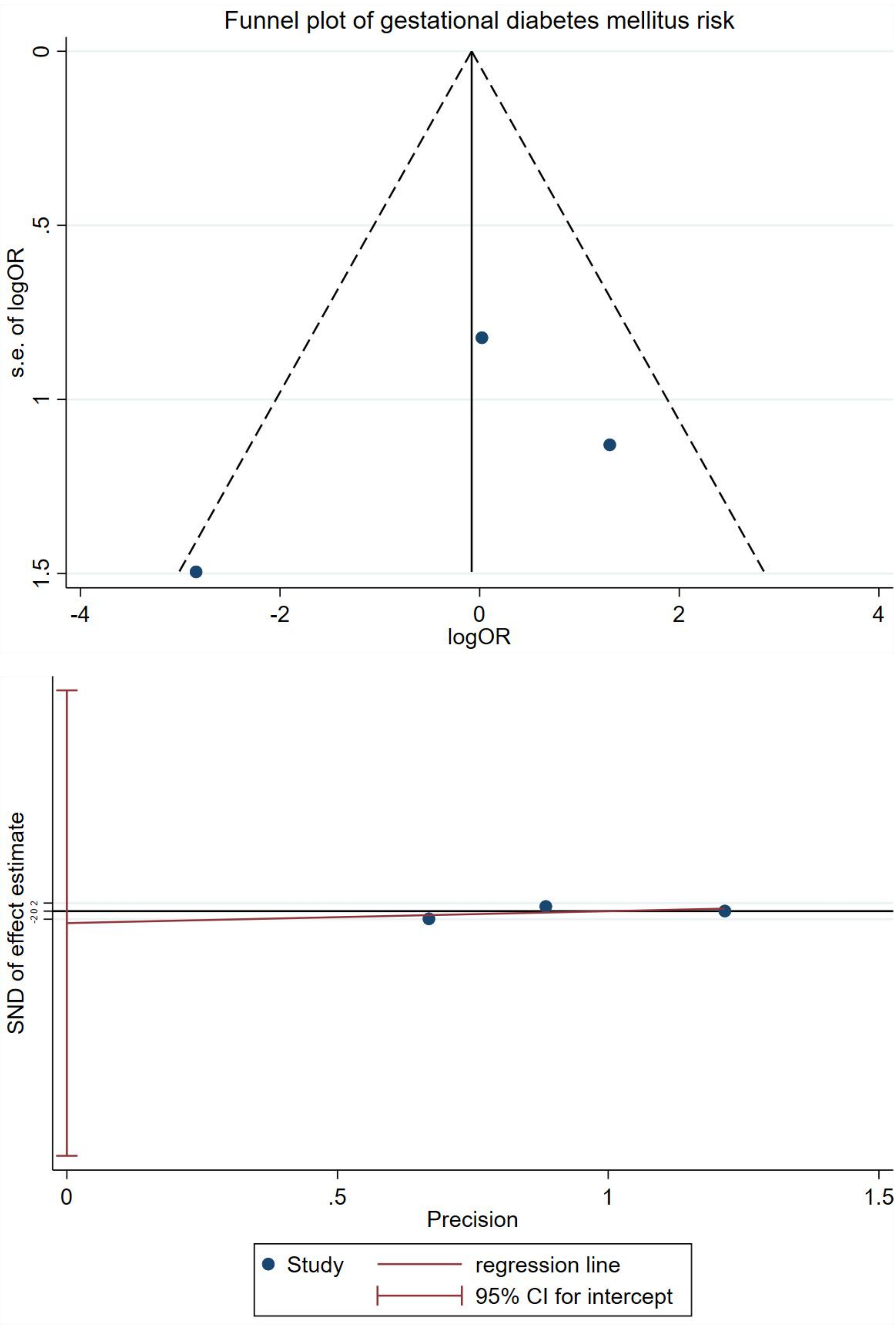
Funnel plot of gestational diabetes mellitus risk
s.e. of logOR
0
.5
1
1.5
-4
-2
0
2
4
logOR
SND of effect estimate
0
.5
1
1.5
Precision
Study
regression line
95% CI for intercept
