# Supplementary material for: Effect of hydroxychloroquine on pregnancy outcome in patients with SLE: a systematic review and meta-analysis
Source: Lupus Sci Med. 2024 Oct 30;11(2):e001239. doi: 10.1136/lupus-2024-001239 (PMC11529578; doi:10.1136/lupus-2024-001239)
Supplement: online supplemental file 5 [file lupus-11-2-s005.pdf]

Supplementary material E: Characteristics of included studies

Study	Year	Type	Baseline comparability	Group	disease duration(year) HG	disease duration(year) CG	Age(year) HG	Age(year) CG	Intervention HG	Intervention CG	HCQ dose (mg/day)	Outcomes	Concurrent medications
Abd et al.[32]	2020	Cohort	YES	HG(47) NG(35)	7.06±4.24	6.57±3.56	32.03±3.92	31.50±3.54	HCQ	NS	NS	1234	COR,AZA, CsA
Canti et al.[14]	2021	Cohort	YES	HG(45) NG(29)	9±1	7±6	32.71±4.36	32.03±4.37	HCQ	NS	300	1234	ASA,LMW-H,AZA,COR
Clowse et al.[33]	2006	Cohort	YES	HG(56) NG(163)	NS				HCQ	NS	NS	123	PDN,AZA
Do et al.[34]	2020	Cohort	YES	HG(53) NG(76)	9.2±5.3	7.2±6.8	33.0±5.5	32.1±6.0	HCQ	NS	NS	1234	ASA,PDN, AZA,Antico-agulant
Kroese et al.[35]	2017	Cohort	NS	HG(30) NG(80)	4.5(3,10.8)	6(3.5,9.5)	32.5±4.3	30.6±4.0	HCQ	NS	200/400	234	NS
Leroux	2015	Cohort	YES	HG(41)	NS		31.2±5.7	30.0±5.4	HCQ	NS	400	1234	PDN,ASA,

et al.[16]				NG(77)									LMWH, AZA,IVIG
Seo et al.[36]	2019	Cohort	YES	HG(80) NG(71)	NS		32.8±4.2	31.8±3.6	HCQ	NS	NS	1234	AZA,COR,A-SA,LMWH
Cao ZJ et al.[37]	2012	Cohort	NS	HG(11) NG(12)	5.6(0.25-12)	5.6(0.25-12)	28.4(22-35)	28.4(22-35)	HCQ+ PDN	PDN	200	123	PDN
Chen GL.[38]	2021	Case-control	YES	HG(137) NG(40)	NS		29.6±4.2	29.9±4.3	HCQ	NS	NS	234	ASA,LMW-H,COR, Immunosupp-ressants
Deng RR.[39]	2017	Case-control	NS	HG(17) NG(67)	4.89±3.82	4.89±3.82	29.89±4.27	29.89±4.27	HCQ	NS	NS	2	COR,ASA, LMWH
Diao MX.[40]	2016	Cohort	YES	HG(34) NG(34)	4.8±2.6	4.8±2.6	25.8±3.3	25.8±3.3	HCQ+ PDN	PDN	400	2	PDN
Wu FQ et al.[41]	2019	Cohort	YES	HG(41) NG(41)	3.2±1.2	3.3±1.4	27.6±5.3	28.6±4.2	HCQ+ PDN	PDN	400	12	PDN, Immunosupp

													-ressants
Lin QR et al.[42]	2021	Cohort	YES	HG(34) NG(34)	3.50±1.09	3.65±1.11	29.45±6.50	28.35±6.42	HCQ+ PDN	PDN	400	12	PDN
Liu XY et al.[43]	2015	Cohort	YES	HG(30) NG(30)	5.4(2-12)	5.4(2-12)	27.5(22-35)	27.5(22-35)	HCQ+ PDN	PDN	200-400	12	PDN
Liu YQ et al.[44]	2013	Cohort	YES	HG(20) NG(20)	2-10	2-10	20-33	20-33	HCQ+ PDN	PDN	200-400	123	PDN
Zheng CS et al.[45]	2015	Cohort	YES	HG(26) NG(26)	3.7±1.4	4.9±1.8	28.4±7.3	26.3±6.1	HCQ+ PDN	PDN	200-400	123	PDN
Wang LH et al.[46]	2018	Cohort	YES	HG(35) NG(35)	5.7±4.3	5.6±4.1	28.5±7.3	28.4±7.1	HCQ+ PDN	PDN	200-400	123	PDN
Wang WJ et al.[47]	2018	Cohort	YES	HG(46) NG(46)	5.4±0.8	5.4±0.7	29.2±4.2	29.7±4.4	HCQ+ PDN	PDN	400	123	PDN

Wu MY.[48]	2014	Case-control	YES	HG(43) NG(40)	5.73±3.23	6.35±4.23	27.60±4.40	27.75±3.89	HCQ+ PDN	PDN	NS	1234	AZA,CsA, PDN
Zhang HQ.[49]	2019	Cohort	YES	HG(32) NG(32)	NS		29.6±2.8	28.4±2.5	HCQ+ PDN	PDN	400	23	PDN, Immunosupp -ressants
Zhou L et al.[50]	2017	Case-control	NS	HG(71) NG(43)	5.2±2.8	5.2±2.8	28±4	28±4	HCQ+ PDN	PDN	200	12	PDN

NS **Not stated;** ***HG*** **HCQ group;** ***NG*** **non-HCQ group;** ***PDN*** **Prednisone;** ***1*** **SLE activity: flare, SLEDAI;** ***2*** **Pregnancy outcome: full-term birth, preterm birth, miscarriage, stillbirth;** ***3*** **Fetal outcome: fetal distress, IUGR, low birth weight, SGA;** ***4*** **Complications of pregnancy: Gestational hypertension, Pre-eclampsia, Gestational diabetes mellitus;** ***COR*** **Corticosteroids;** ***AZA*** **Azathioprine;** ***CsA*** **Cyclosporine A;** ***ASA*** **Aspirin;** ***LMWH*** **Low molecular weight heparin;** ***IVIG*** **Intravenous immunoglobulin.**
