# Supplementary material for: Effect of hydroxychloroquine on pregnancy outcome in patients with SLE: a systematic review and meta-analysis
Source: Lupus Sci Med. 2024 Oct 30;11(2):e001239. doi: 10.1136/lupus-2024-001239 (PMC11529578; doi:10.1136/lupus-2024-001239)

Supplementary material F: Forest plots

F.1. Pooled OR of flare risk

Study or Subgroup	HCQ Events	HCQ Total	non-HCQ Events	non-HCQ Total	Weight	Odds Ratio M-H, Random, 95% CI
Abd et al.,2020	1	47	2	35	3.9%	0.36 [0.03, 4.12]
Clowse et al.,2006	17	56	59	163	15.5%	0.77 [0.40, 1.48]
Do et al.,2020	12	53	18	76	13.5%	0.94 [0.41, 2.17]
Leroux et al.,2015	7	41	20	77	12.2%	0.59 [0.22, 1.53]
Liu YQ et al.,2013	6	20	12	20	9.1%	0.29 [0.08, 1.06]
Seo et al.,2019	20	80	8	71	12.9%	2.63 [1.07, 6.41]
Wu MY.,2014	4	43	13	40	9.8%	0.21 [0.06, 0.72]
Zheng CS et al.,2015	7	26	14	26	10.3%	0.32 [0.10, 1.01]
Zhou L et al.,2017	11	71	16	43	12.9%	0.31 [0.13, 0.75]
**Total (95% CI)**		**437**		**551**	**100.0%**	**0.57 [0.33, 0.97]**
Total events	85		162			

Heterogeneity: $Tau^2 = 0.37$; $Chi^2 = 19.52$, df = 8 (P = 0.01); $I^2 = 59\%$
Test for overall effect: Z = 2.07 (P = 0.04)

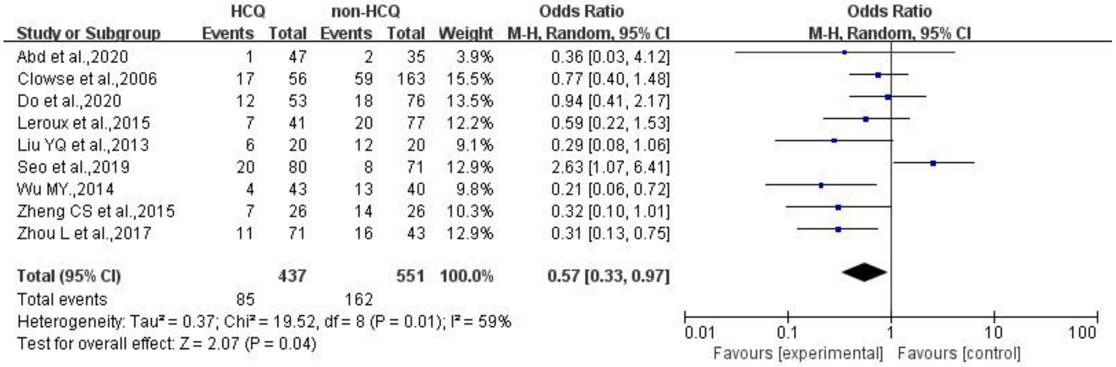

F.2. Pooled MD of SLEDAI risk during the first trimester

Study or Subgroup	HCQ Mean	HCQ SD	HCQ Total	non-HCQ Mean	non-HCQ SD	non-HCQ Total	Weight	Mean Difference IV, Fixed, 95% CI
Canti et al., 2021	2.24	2.28	45	1.72	2.83	29	9.7%	0.52 [-0.71, 1.75]
Cao ZJ et al., 2012	7.82	2.14	11	7.83	2.17	12	4.7%	-0.01 [-1.77, 1.75]
Lin QR et al.,2021	8.01	1.95	34	7.98	2	34	16.6%	0.03 [-0.91, 0.97]
Liu XY et al.,2015	7.83	2.11	30	8.02	2.23	30	12.1%	-0.19 [-1.29, 0.91]
Wang LH et al.,2018	8	2.3	35	8.1	2.2	35	13.2%	-0.10 [-1.15, 0.95]
Wang WJ et al.,2018	8	1.7	46	8.2	1.8	46	28.6%	-0.20 [-0.92, 0.52]
Wu FQ et al.,2019	7.91	2.31	41	8.01	2.26	41	15.0%	-0.10 [-1.09, 0.89]
**Total (95% CI)**			**242**			**227**	**100.0%**	**-0.05 [-0.44, 0.33]**

Heterogeneity: $Chi^2 = 1.11$, df = 6 (P = 0.98); $I^2 = 0\%$
Test for overall effect: Z = 0.27 (P = 0.78)

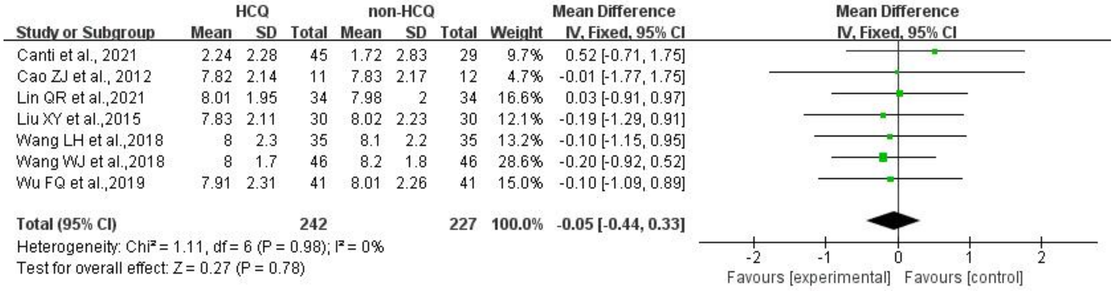

F.3. Pooled MD of SLEDAI risk during the second trimester

Study or Subgroup	HCQ Mean	HCQ SD	HCQ Total	non-HCQ Mean	non-HCQ SD	non-HCQ Total	Weight	Mean Difference IV, Random, 95% CI
Canti et al., 2021	2	2.43	45	1.61	2.48	29	13.9%	0.39 [-0.76, 1.54]
Cao ZJ et al., 2012	5.55	2.11	11	7.67	2.61	12	7.9%	-2.12 [-4.05, -0.19]
Lin QR et al.,2021	5.13	1.66	34	7.42	1.98	34	16.9%	-2.29 [-3.16, -1.42]
Liu XY et al.,2015	5.32	2.03	30	7.61	2.67	30	13.4%	-2.29 [-3.49, -1.09]
Wang LH et al.,2018	5.4	2.5	35	7.5	2.3	35	14.1%	-2.10 [-3.23, -0.97]
Wang WJ et al.,2018	5.3	1.1	46	7.2	1.6	46	20.3%	-1.90 [-2.46, -1.34]
Wu FQ et al.,2019	5.34	2.08	41	7.61	3.23	41	13.6%	-2.27 [-3.45, -1.09]
**Total (95% CI)**			**242**			**227**	**100.0%**	**-1.80 [-2.46, -1.13]**

Heterogeneity: $Tau^2 = 0.48$; $Chi^2 = 16.90$, df = 6 (P = 0.010); $I^2 = 64\%$
Test for overall effect: Z = 5.31 (P < 0.00001)

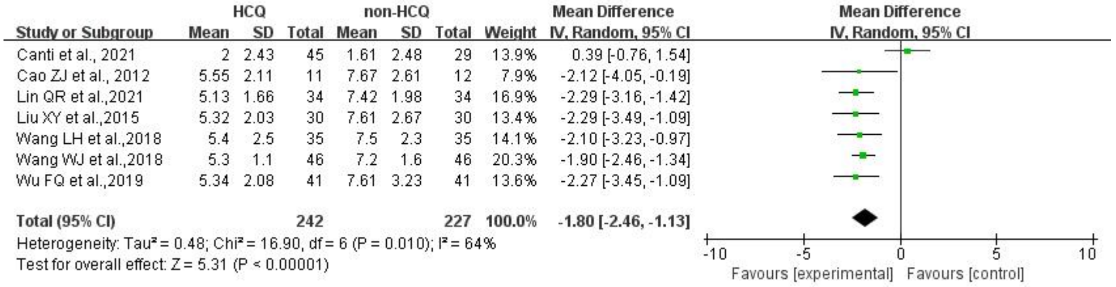

F.4. Pooled MD of SLEDAI risk during the third trimester

Study or Subgroup	HCQ Mean	HCQ SD	HCQ Total	non-HCQ Mean	non-HCQ SD	non-HCQ Total	Weight	Mean Difference IV, Random, 95% CI
Canti et al., 2021	2.03	2.71	45	1.24	1.7	29	14.8%	0.79 [-0.21, 1.79]
Cao ZJ et al., 2012	4.18	2.23	11	6.5	2.81	12	10.1%	-2.32 [-4.39, -0.25]
Liu XY et al.,2015	3.33	2.26	30	6.03	2.89	30	13.4%	-2.70 [-4.01, -1.39]
Liu YQ et al.,2013	3.12	1.03	34	6.81	1.95	34	15.8%	-3.69 [-4.43, -2.95]
Wang LH et al.,2018	3.4	2.3	35	5.9	2.6	35	14.1%	-2.50 [-3.65, -1.35]
Wang WJ et al.,2018	3.3	0.6	46	5.9	1.2	46	16.9%	-2.60 [-2.99, -2.21]
Wu FQ et al.,2019	3.21	1.67	41	6.21	2.76	41	14.9%	-3.00 [-3.99, -2.01]
**Total (95% CI)**			**242**			**227**	**100.0%**	**-2.30 [-3.31, -1.29]**

Heterogeneity: $Tau^2 = 1.54$; $Chi^2 = 52.43$, df = 6 (P < 0.00001); $I^2 = 89\%$
Test for overall effect: Z = 4.46 (P < 0.00001)

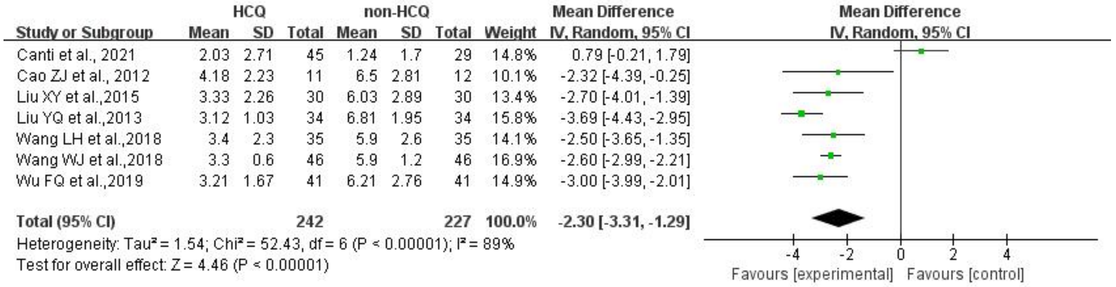

F.5. Pooled OR of full-term birth risk

Study or Subgroup	HCQ Events	HCQ Total	non-HCQ Events	non-HCQ Total	Weight	Odds Ratio M-H, Fixed, 95% CI
Abd et al.,2020	26	47	14	35	10.1%	1.86 [0.76, 4.51]
Cao ZJ et al., 2012	9	11	8	12	2.0%	2.25 [0.32, 15.76]
Clowse et al.,2006	30	56	93	163	31.0%	0.87 [0.47, 1.60]
Deng RR.,2017	11	17	39	67	7.8%	1.32 [0.44, 3.98]
Diao MX.,2016	23	34	15	34	6.8%	2.65 [0.99, 7.11]
Kroese et al.,2017	18	30	52	80	15.9%	0.81 [0.34, 1.91]
Lin QR et al.,2021	30	34	22	34	3.6%	4.09 [1.16, 14.40]
Liu XY et al.,2015	26	30	19	30	3.6%	3.76 [1.04, 13.65]
Liu YQ et al.,2013	17	20	10	20	2.1%	5.67 [1.25, 25.61]
Wang LH et al.,2018	30	35	21	35	4.2%	4.00 [1.25, 12.80]
Wang WJ et al.,2018	41	46	32	46	4.9%	3.59 [1.17, 11.01]
Wu FQ et al.,2019	35	41	23	41	4.7%	4.57 [1.58, 13.22]
Zheng CS et al.,2015	22	26	15	26	3.2%	4.03 [1.08, 15.09]
**Total (95% CI)**		**427**		**623**	**100.0%**	**2.01 [1.52, 2.65]**
Total events	318		363			

Heterogeneity: Chi² = 22.14, df = 12 (P = 0.04); I² = 46%
Test for overall effect: Z = 4.88 (P < 0.00001)

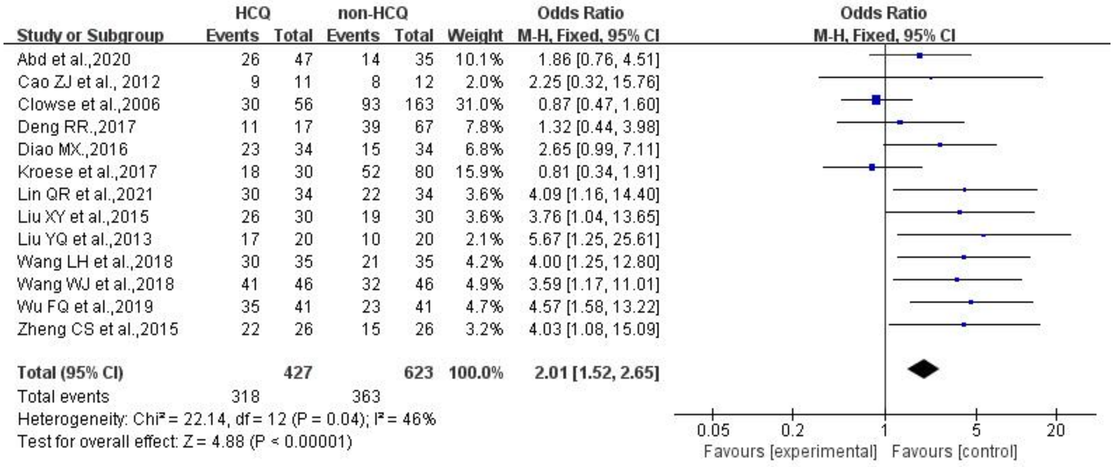

F.6. Pooled OR of preterm birth risk

Study or Subgroup	HCQ Events	HCQ Total	non-HCQ Events	non-HCQ Total	Weight	Odds Ratio M-H, Fixed, 95% CI
Abd et al.,2020	11	47	14	35	6.4%	0.46 [0.18, 1.19]
Canti et al., 2021	7	45	7	29	3.7%	0.58 [0.18, 1.87]
Cao ZJ et al., 2012	1	11	2	12	0.9%	0.50 [0.04, 6.44]
Chen GL.,2021	32	137	4	40	2.5%	2.74 [0.91, 8.29]
Clowse et al.,2006	19	56	64	163	11.2%	0.79 [0.42, 1.50]
Deng RR.,2017	5	17	18	67	2.7%	1.13 [0.35, 3.67]
Diao MX.,2016	10	34	15	34	5.5%	0.53 [0.19, 1.44]
Do et al.,2020	18	53	31	76	8.7%	0.75 [0.36, 1.55]
Kroese et al.,2017	2	30	16	80	4.2%	0.29 [0.06, 1.33]
Leroux et al.,2015	6	41	36	77	11.0%	0.20 [0.07, 0.52]
Lin QR et al.,2021	3	34	10	34	4.7%	0.23 [0.06, 0.94]
Liu XY et al.,2015	2	30	5	30	2.4%	0.36 [0.06, 2.01]
Liu YQ et al.,2013	2	20	8	20	3.7%	0.17 [0.03, 0.92]
Seo et al.,2019	25	80	35	71	13.2%	0.47 [0.24, 0.91]
Wang LH et al.,2018	3	35	6	35	2.8%	0.45 [0.10, 1.98]
Wang WJ et al.,2018	3	46	9	46	4.3%	0.29 [0.07, 1.14]
Wu FQ et al.,2019	2	41	7	41	3.4%	0.25 [0.05, 1.28]
Wu MY.,2014	6	43	8	40	3.7%	0.65 [0.20, 2.07]
Zhang HQ et al.,2019	1	32	5	32	2.5%	0.17 [0.02, 1.58]
Zhou L et al.,2017	16	71	5	43	2.5%	2.21 [0.75, 6.55]
**Total (95% CI)**		**903**		**1005**	**100.0%**	**0.57 [0.46, 0.72]**
Total events	174		305			

Heterogeneity: Chi² = 29.65, df = 19 (P = 0.06); I² = 36%
Test for overall effect: Z = 4.73 (P < 0.00001)

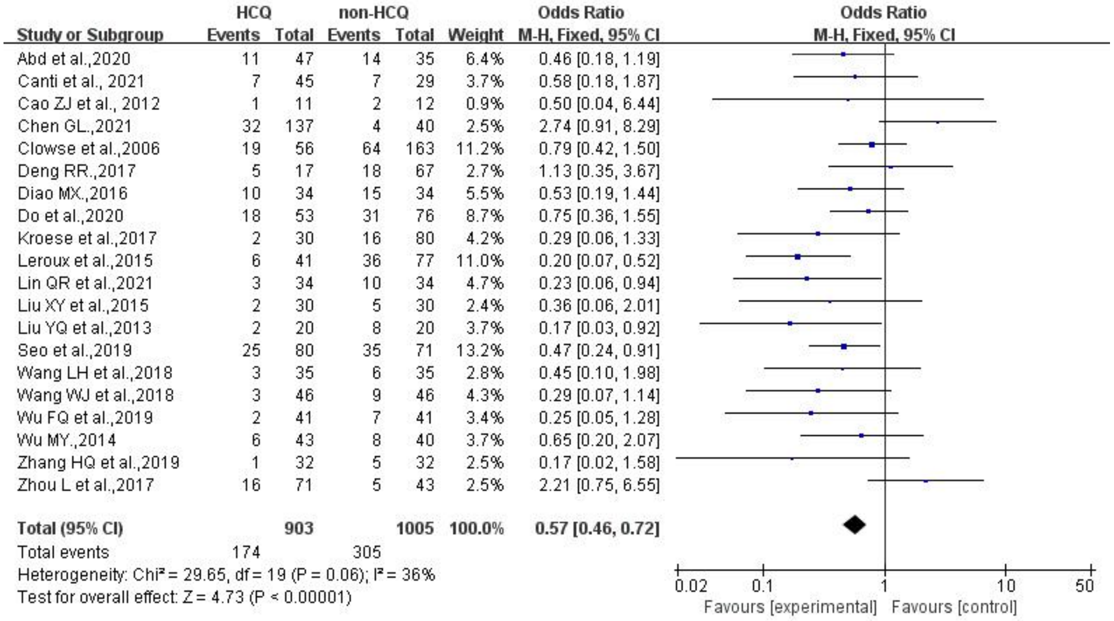

F.7. Pooled OR of miscarriage risk

Study or Subgroup	HCQ Events	HCQ Total	non-HCQ Events	non-HCQ Total	Weight	Odds Ratio M-H, Fixed, 95% CI
Abd et al.,2020	10	47	7	35	20.0%	1.08 [0.37, 3.19]
Canti et al., 2021	6	45	4	29	13.3%	0.96 [0.25, 3.75]
Chen GL.,2021	4	137	1	40	4.7%	1.17 [0.13, 10.80]
Clowse et al.,2006	7	56	6	163	8.5%	3.74 [1.20, 11.65]
Kroese et al.,2017	9	30	10	80	12.1%	3.00 [1.08, 8.35]
Seo et al.,2019	4	80	4	71	12.7%	0.88 [0.21, 3.66]
Wu MY.,2014	1	43	3	40	9.6%	0.29 [0.03, 2.95]
Zhou L et al.,2017	2	71	5	43	19.1%	0.22 [0.04, 1.19]
**Total (95% CI)**		**509**		**501**	**100.0%**	**1.26 [0.79, 2.01]**
Total events	43		40			

Heterogeneity: Chi² = 12.38, df = 7 (P = 0.09); I² = 43%
Test for overall effect: Z = 0.98 (P = 0.33)

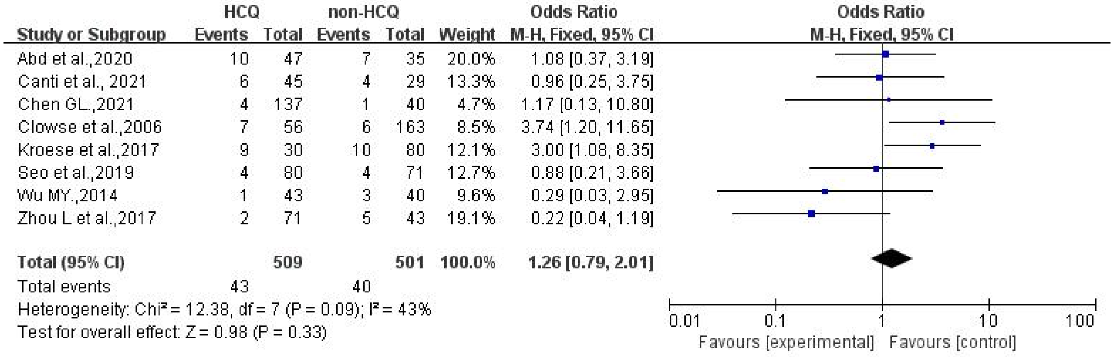

F.8. Pooled OR of stillbirth risk

Study or Subgroup	HCQ Events	HCQ Total	non-HCQ Events	non-HCQ Total	Weight	Odds Ratio M-H, Fixed, 95% CI
Canti et al., 2021	0	45	1	29	7.1%	0.21 [0.01, 5.30]
Chen GL.,2021	6	137	2	40	11.7%	0.87 [0.17, 4.49]
Clowse et al.,2006	3	56	13	163	24.8%	0.65 [0.18, 2.38]
Kroese et al.,2017	1	30	2	80	4.2%	1.34 [0.12, 15.40]
Leroux et al.,2015	3	41	3	77	7.6%	1.95 [0.37, 10.11]
Seo et al.,2019	4	80	3	71	11.9%	1.19 [0.26, 5.52]
Wu MY.,2014	4	43	2	40	7.4%	1.95 [0.34, 11.27]
Zhang HQ et al.,2019	0	32	1	32	5.8%	0.32 [0.01, 8.23]
Zhou L et al.,2017	1	71	4	43	19.4%	0.14 [0.02, 1.29]
**Total (95% CI)**		**535**		**575**	**100.0%**	**0.82 [0.46, 1.46]**
Total events	22		31			

Heterogeneity: Chi² = 5.95, df = 8 (P = 0.65); I² = 0%
Test for overall effect: Z = 0.68 (P = 0.49)

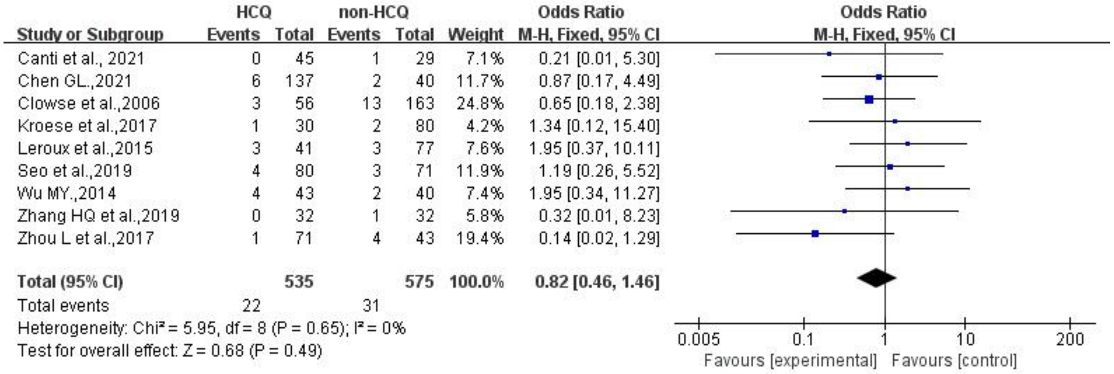

F.9. Pooled OR of fetal distress risk

Study or Subgroup	HCQ Events	HCQ Total	non-HCQ Events	non-HCQ Total	Weight	Odds Ratio M-H, Fixed, 95% CI
Abd et al.,2020	7	47	2	35	10.4%	2.89 [0.56, 14.85]
Canti et al., 2021	1	45	3	29	19.1%	0.20 [0.02, 1.99]
Chen GL.,2021	14	137	6	40	44.6%	0.64 [0.23, 1.80]
Wu MY.,2014	3	43	1	40	5.2%	2.92 [0.29, 29.34]
Zhang HQ et al.,2019	1	32	4	32	20.7%	0.23 [0.02, 2.14]
**Total (95% CI)**		**304**		**176**	**100.0%**	**0.82 [0.42, 1.61]**
Total events	26		16			

Heterogeneity: Chi² = 6.37, df = 4 (P = 0.17); I² = 37%
Test for overall effect: Z = 0.57 (P = 0.57)

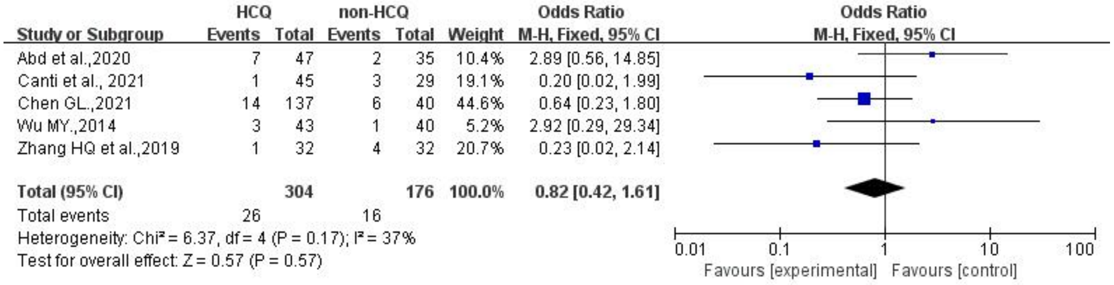

F.10. Pooled OR of IUGR risk

Study or Subgroup	HCQ Events	HCQ Total	non-HCQ Events	non-HCQ Total	Weight	Odds Ratio M-H, Fixed, 95% CI
Abd et al.,2020	6	47	10	35	15.6%	0.37 [0.12, 1.13]
Canti et al., 2021	4	45	8	29	13.9%	0.26 [0.07, 0.95]
Cao ZJ et al., 2012	1	11	3	12	4.1%	0.30 [0.03, 3.43]
Chen GL.,2021	9	137	4	40	9.0%	0.63 [0.18, 2.17]
Do et al.,2020	9	53	3	76	3.2%	4.98 [1.28, 19.38]
Leroux et al.,2015	4	41	33	77	32.3%	0.14 [0.05, 0.44]
Liu YQ et al.,2013	1	20	3	20	4.5%	0.30 [0.03, 3.15]
Wang LH et al.,2018	1	35	2	35	3.0%	0.49 [0.04, 5.61]
Wu MY.,2014	2	43	1	40	1.5%	1.90 [0.17, 21.83]
Zhang HQ et al.,2019	1	32	3	32	4.5%	0.31 [0.03, 3.17]
Zheng CS et al.,2015	3	26	6	26	8.3%	0.43 [0.10, 1.97]
**Total (95% CI)**		**490**		**422**	**100.0%**	**0.48 [0.31, 0.72]**
Total events	41		76			

Heterogeneity: Chi² = 18.72, df = 10 (P = 0.04); I² = 47%
Test for overall effect: Z = 3.46 (P = 0.0005)

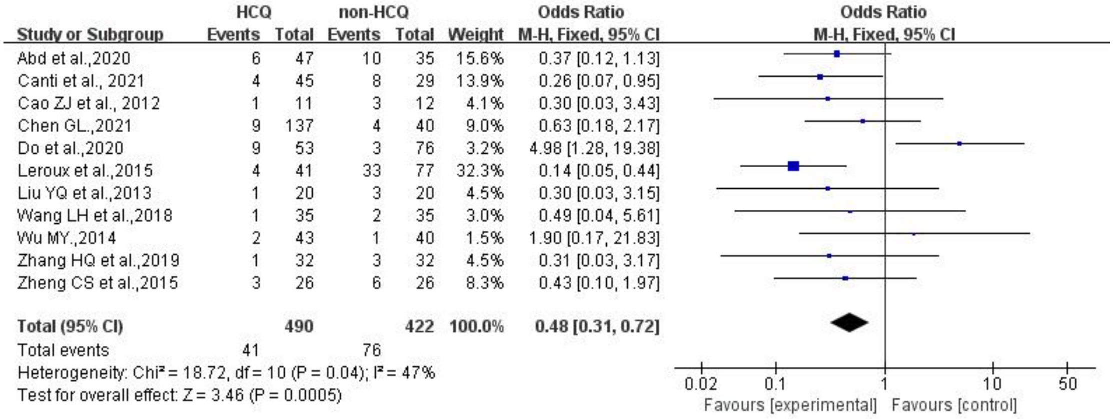

F.11. Pooled OR of low birth weight risk

Study or Subgroup	HCQ Events	HCQ Total	non-HCQ Events	non-HCQ Total	Weight	Odds Ratio M-H, Random, 95% CI
Chen GL.,2021	29	137	6	40	28.4%	1.52 [0.58, 3.97]
Wang WJ et al.,2018	2	46	6	46	21.2%	0.30 [0.06, 1.59]
Wu MY.,2014	4	43	6	40	24.4%	0.58 [0.15, 2.23]
Zhang HQ et al.,2019	5	32	20	32	26.0%	0.11 [0.03, 0.37]
**Total (95% CI)**		**258**		**158**	**100.0%**	**0.43 [0.13, 1.50]**
Total events	40		38			

Heterogeneity: Tau² = 1.17; Chi² = 11.67, df = 3 (P = 0.009); I² = 74%
Test for overall effect: Z = 1.32 (P = 0.19)

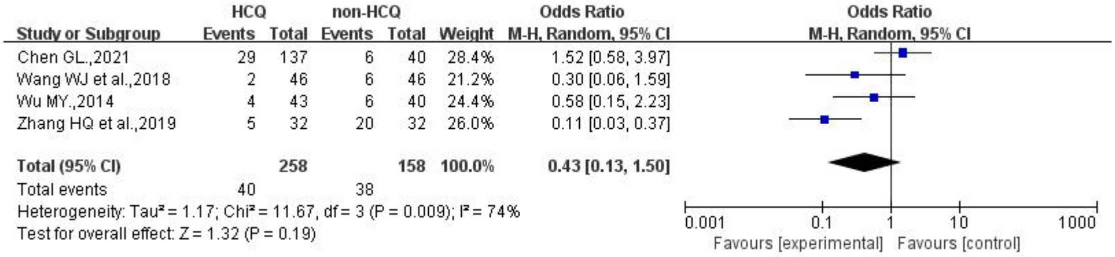

F.12. Pooled OR of SGA risk

Study or Subgroup	HCQ Events	HCQ Total	non-HCQ Events	non-HCQ Total	Weight	Odds Ratio M-H, Fixed, 95% CI
Clowse et al.,2006	11	56	29	163	33.3%	1.13 [0.52, 2.44]
Kroese et al.,2017	5	30	10	80	12.7%	1.40 [0.44, 4.50]
Seo et al.,2019	24	80	26	71	53.9%	0.74 [0.38, 1.46]
**Total (95% CI)**		**166**		**314**	**100.0%**	**0.95 [0.60, 1.53]**
Total events	40		65			

Heterogeneity: $Chi^2 = 1.13$, df = 2 (P = 0.57); $I^2 = 0\%$
Test for overall effect: Z = 0.19 (P = 0.85)

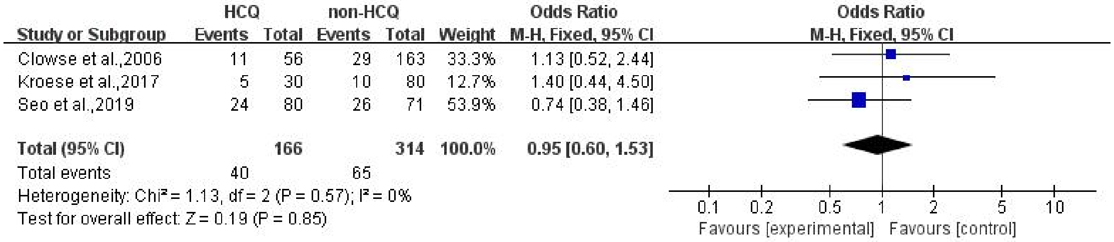

F.13. Pooled OR of gestational hypertension risk

Study or Subgroup	HCQ Events	HCQ Total	non-HCQ Events	non-HCQ Total	Weight	Odds Ratio M-H, Fixed, 95% CI
Abd et al.,2020	3	47	15	35	52.6%	0.09 [0.02, 0.35]
Canti et al., 2021	5	45	5	29	17.6%	0.60 [0.16, 2.29]
Leroux et al.,2015	0	41	9	77	21.5%	0.09 [0.00, 1.53]
Wu MY.,2014	0	43	2	40	8.4%	0.18 [0.01, 3.80]
**Total (95% CI)**		**176**		**181**	**100.0%**	**0.19 [0.08, 0.42]**
Total events	8		31			

Heterogeneity: $Chi^2 = 4.29$, df = 3 (P = 0.23); $I^2 = 30\%$
Test for overall effect: Z = 4.02 (P < 0.0001)

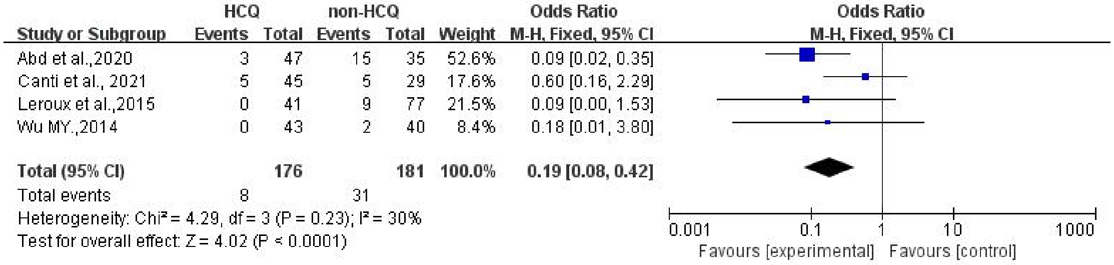

F.14. Pooled OR of pre-eclampsia risk

Study or Subgroup	HCQ Events	HCQ Total	non-HCQ Events	non-HCQ Total	Weight	Odds Ratio M-H, Fixed, 95% CI
Abd et al.,2020	2	47	5	35	9.7%	0.27 [0.05, 1.47]
Canti et al., 2021	1	45	4	29	8.4%	0.14 [0.02, 1.34]
Chen GL.,2021	15	137	6	40	14.7%	0.70 [0.25, 1.93]
Do et al.,2020	7	53	20	76	25.3%	0.43 [0.17, 1.10]
Kroese et al.,2017	2	30	9	80	8.1%	0.56 [0.11, 2.77]
Leroux et al.,2015	3	41	5	77	5.7%	1.14 [0.26, 5.02]
Seo et al.,2019	6	80	14	71	24.4%	0.33 [0.12, 0.91]
Wu MY.,2014	1	43	2	40	3.6%	0.45 [0.04, 5.19]
**Total (95% CI)**		**476**		**448**	**100.0%**	**0.46 [0.29, 0.72]**
Total events	37		65			

Heterogeneity: $Chi^2 = 4.01$, df = 7 (P = 0.78); $I^2 = 0\%$
Test for overall effect: Z = 3.37 (P = 0.0008)

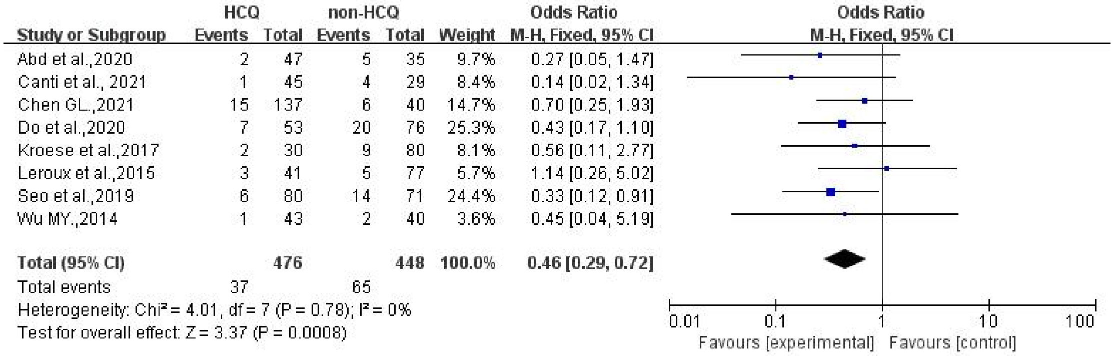

F.15. Pooled OR of gestational diabetes mellitus risk

Study or Subgroup	HCQ Events	HCQ Total	non-HCQ Events	non-HCQ Total	Weight	Odds Ratio M-H, Random, 95% CI
Abd et al.,2020	0	47	5	35	25.7%	0.06 [0.00, 1.09]
Chen GL.,2021	7	137	2	40	40.9%	1.02 [0.20, 5.13]
Seo et al.,2019	4	80	1	71	33.3%	3.68 [0.40, 33.76]
**Total (95% CI)**		**264**		**146**	**100.0%**	**0.75 [0.10, 5.75]**
Total events	11		8			

Heterogeneity: $Tau^2 = 1.96$; $Chi^2 = 5.14$, df = 2 (P = 0.08); $I^2 = 61\%$
Test for overall effect: Z = 0.28 (P = 0.78)

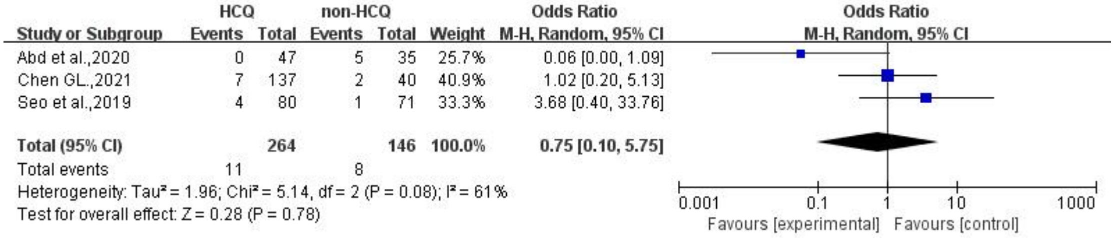

Supplement: online supplemental file 6 [file lupus-11-2-s006.pdf]
